# Reduced oxycodone brain delivery in rats due to lipopolysaccharide-induced inflammation: microdialysis insights into brain disposition and sex-specific pharmacokinetics

**DOI:** 10.1186/s12987-024-00598-6

**Published:** 2024-12-02

**Authors:** Frida Bällgren, Margareta Hammarlund-Udenaes, Irena Loryan

**Affiliations:** Translational Pharmacokinetics/Pharmacodynamics Group (tPKPD), Department of Pharmacy, Uppsala University, Box 580, 75123 Uppsala, Sweden

**Keywords:** Blood–brain barrier, Blood-cerebrospinal fluid barrier, Oxycodone, Microdialysis, Sex, Proton-coupled organic cation (H^+^/OC) antiporter, Lipopolysaccharides, Inflammation, Proteomics

## Abstract

**Background:**

Oxycodone, a widely used opioid analgesic, has an unbound brain-to-plasma concentration ratio (K_p,uu_) greater than unity, indicating active uptake across brain barriers associated with the putative proton-coupled organic cation (H^+^/OC) antiporter system. With this study, we aimed to elucidate oxycodone's CNS disposition during lipopolysaccharide (LPS)-induced systemic inflammation in Sprague–Dawley rats.

**Methods:**

Using brain microdialysis, we dynamically and simultaneously monitored unbound oxycodone concentrations in blood, striatum, lateral ventricle, and *cisterna magna* following intravenous administration of oxycodone post-LPS challenge.

**Results:**

Our results indicated a reduced, sex-independent brain net uptake of oxycodone across the blood–brain barrier (BBB) measured in the striatum. Notably, the LPS challenge has significantly altered the systemic pharmacokinetics (PK) of oxycodone, in a sex-specific manner, leading to lower clearance and higher blood concentrations in females compared to LPS-treated males and healthy rats of both sexes. Proteomic analysis using Olink Target 96 Mouse Exploratory assay confirmed the induction of systemic inflammation and neuroinflammation. The inflammation led to an increased paracellular transport, measured using 4 kDa dextran, while preserving net active uptake of oxycodone across both BBB and the blood-cerebrospinal fluid barrier (BCSFB), with K_p,uu_ values of 2.7 and 2.5, respectively. The extent of uptake was 1.6-fold lower (p < 0.0001) at the BBB and unchanged at the BCSFB after the LPS challenge compared to that in healthy rats. However, the mean exposure of unbound oxycodone in the brain following LPS was similar to that in healthy rats, primarily due to the LPS-induced changes in systemic exposure.

**Conclusions:**

These findings highlight the dissimilar responses at blood–brain interfaces during LPS-induced inflammation. Advancing the knowledge of neuropharmacokinetic mechanisms, specifically those involving the H^+^/OC antiporter system, will enable the development of more effective therapeutic strategies during inflammation conditions.

**Graphical Abstract:**

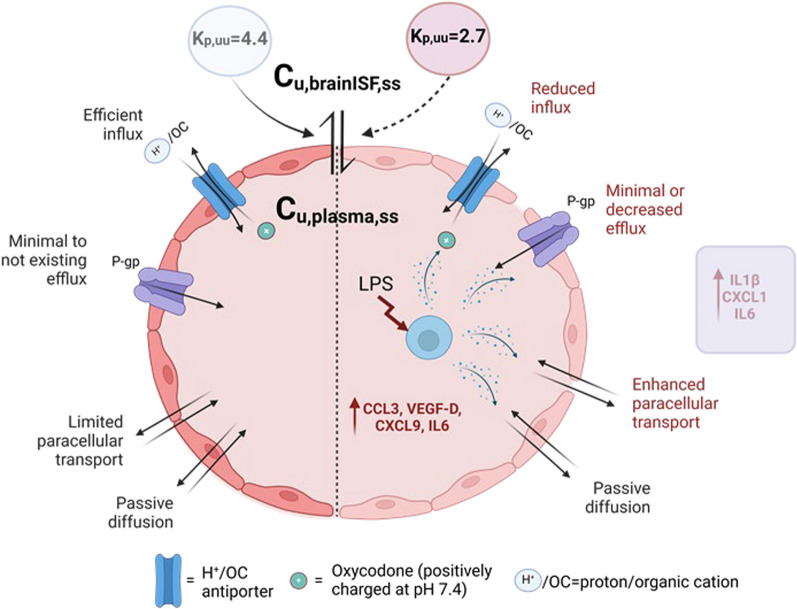

**Supplementary Information:**

The online version contains supplementary material available at 10.1186/s12987-024-00598-6.

## Introduction

The opioid analgesic oxycodone has a unique property of active uptake from blood to brain across the blood–brain barrier (BBB), first identified using brain microdialysis in rats by Boström et al. [1]. Recently, this active uptake was confirmed not only across the BBB but also at the blood-cerebrospinal fluid barrier (BCSFB) [2]. This uptake has been associated with the putative proton-coupled organic cation (H^+^/OC) antiporter system [3, 4], which is known for its contribution to concentration- and pH-dependent drug uptake, and for facilitating the exchange of a proton for an organic cation [5].

The net transport of drugs across brain barriers is a complex and selectively regulated process involving multiple concurrent pathways, including transcellular (passive diffusion, transcytosis, active influx, and efflux) and limited paracellular processes. Active net uptake of oxycodone at the brain barriers is beneficial for its CNS drug delivery and the achievement of sufficient and fast exposure at the target site, to obtain a desired effect at therapeutic doses. Hence, potential pathology-induced changes in the active influx transport of drugs, across the brain barriers may have a crucial role in their therapeutic effect in disease conditions including inflammation.

Endotoxemia, characterized by the presence of endotoxins in the blood, often leads to systemic inflammation and is a critical factor in conditions like sepsis [6]. Bacterial endotoxins, specifically lipopolysaccharides (LPS), are among the most effective stimulators of the immune system and are potent pro-inflammatory agents, known for triggering the release of pro-inflammatory cytokines and acute phase proteins in a species- and LPS origin-dependent manner [7–11]. Clinically, systemic inflammation poses significant challenges due to its impact on various organs, including the brain and brain barriers, by activating the brain’s innate immune response and triggering the expression of a wide range of inflammatory mediators [12, 13]. Immune modulation in the blood and brain represents a complex topic from the standpoint of the anatomical microenvironment involving both blood–brain interfaces and CNS fluids. In a recent study, we explored the CNS delivery of oxycodone in pigs under both healthy and acute endotoxemic conditions, revealing a reduced extent of uptake across brain barriers under endotoxemia [14]. Although this study provided foundational insights into the CNS drug disposition of oxycodone in pigs, it had inevitable limitations. In particular, microdialysis in pigs was conducted under anesthesia and was limited to a short timeframe, corresponding to the acute stage of endotoxemia. In this regard, performing longitudinal microdialysis studies in awake rats allows for extended temporal resolution and the ability to administer repeated doses of LPS. This approach can provide additional insights into potential alterations in oxycodone CNS disposition and potential therapeutic adjustments needed for effective pain management in patients with endotoxemia, with further impact also on other drugs that are actively taken up at the brain barriers.

The extent of CNS drug delivery is commonly evaluated using the unbound partition coefficient, K_p,uu_ metrics [15, 16]. K_p,uu,brain_ is the ratio of unbound drug in brain interstitial fluid (ISF) to that in blood, with values above unity indicating active uptake [17–19]. Similarly, K_p,uu,CSF_ describes the extent of drug delivery to CSF across BCSFB in choroidal plexuses. As the drug concentrations in blood seldom serves as a good surrogate for brain ISF concentrations [15], and because only the unbound drug can initiate a therapeutic effect [20, 21], the estimation of unbound concentration at the target site is critical. This can be achieved through the brain microdialysis technique for collecting dialysate, containing only the unbound drug concentration from the probe placement site. Therefore, microdialysis is a gold standard for dynamic characterization of transport across brain barriers as well as a tool for direct and simultaneous comparison between drug exposure in brain ISF and CSF, allowing validation of CSF as a proxy for assessment of brain exposure [22, 23].

Therefore, we conducted this study to enhance the understanding of oxycodone CNS disposition under disease conditions, with a particular focus on active uptake transport in endotoxemia. Using an LPS-induced inflammation model in female and male rats, we investigated the systemic pharmacokinetics (PK) of oxycodone, its extent of transport across the BBB and BCSFB, and delivery to *cisterna magna* as well as its intra-brain distribution through microdialysis.

## Materials and methods

An extended materials and methods section is presented in Supplementary Materials.

### Animals

Experiments were performed on drug-naïve male and female Sprague–Dawley rats (n = 26) (Taconic, Lille Skensved, Denmark) weighing 270–330 g. The rats were group housed under a 12-h light–dark cycle at a temperature of 20–21 °C and humidity of 45–65% with food and water ad libitum. Guidelines from the Swedish National Board for Laboratory Animals were applied and the Animal Ethics Committee of Uppsala, Sweden, approved the study (Ethical Approval Dnr. 5.8.18–122302019). To ensure transparent and comprehensive reporting, ARRIVE 2.0 guidelines have been applied [24]. Pain and distress levels were evaluated in animals according to Uppsala University guidelines. Given the desired probability level (α = 0.05), effect size (Cohen’s d = 1.2–1.5), and statistical power level (0.8), the sample size per group required for a two-tailed t-test study was estimated to be minimally four to six [25]. The study was not blinded and no randomization was applied.

The impact of the LPS challenge on various PK parameters was compared to the same parameters measured in healthy male and female Sprague–Dawley rats, reported elsewhere [2]. The studies in healthy and LPS-challenged animals were performed during the same period.

### Placement of probes and catheters

**Fig. 1:**
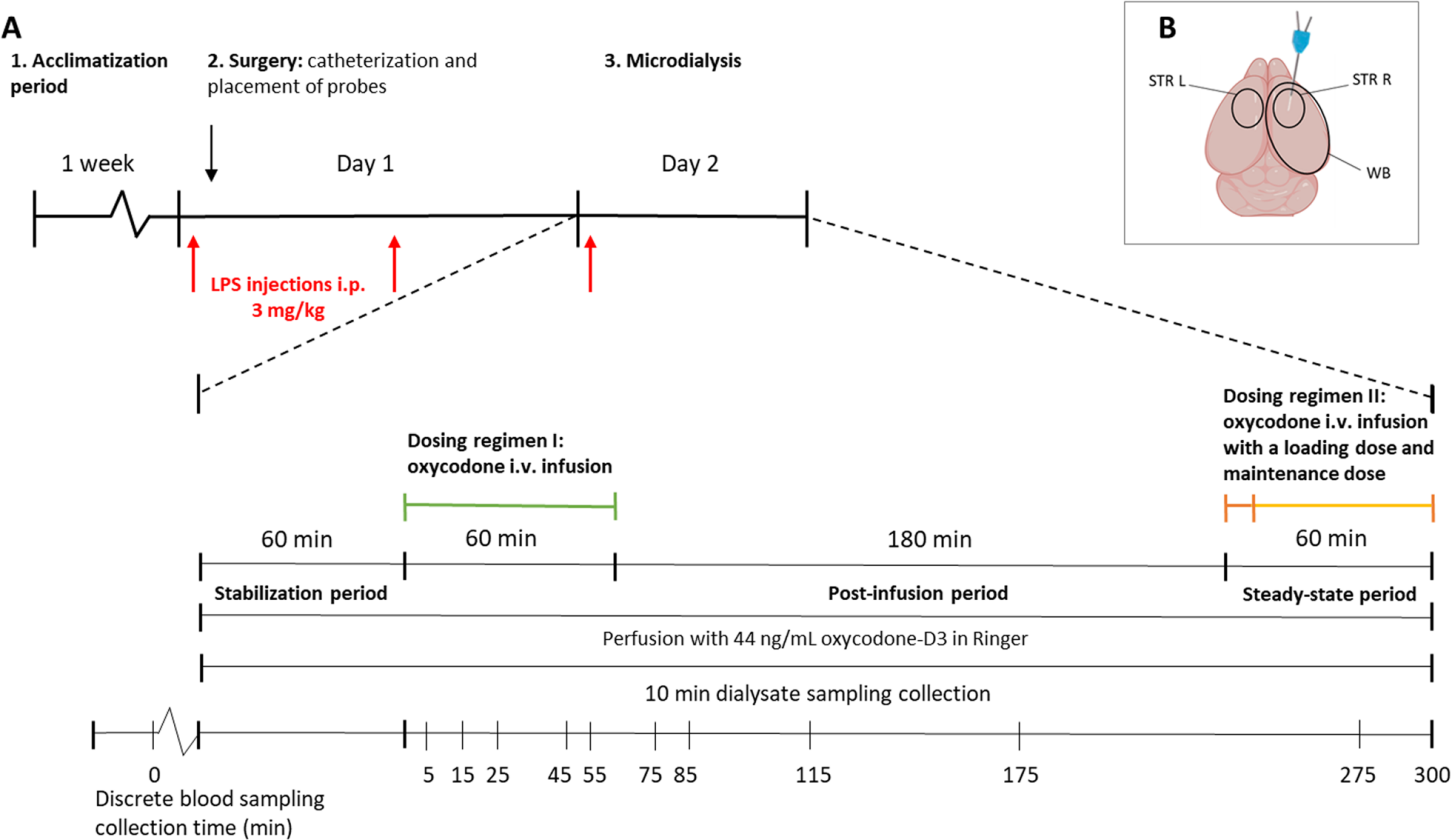
Overview of the experimental design. **A** Rats were acclimatized for one week. Surgery was performed on Day 1, and the microdialysis experiment was performed on Day 2 . LPS was administered on three occasions during Day 1–2. Surgery included catheterization of blood vessels for drug administration, blood sampling and implantation of the CNS and blood probes. The microdialysis experiment was initiated 27 h after the first LPS dose. It included a 60-min stabilization period followed by 300 min of sample collection after two oxycodone administrations (Dosing regimens I and II) which were separated by a washout period (Post-infusion period). Dosing regimen I was a 60-min intravenous (iv) infusion of 0.3 mg/kg/h, with a Post-infusion washout period of 180 min. Dosing regimen II was an iv loading dose of 0.24 mg/kg, administered as an infusion over two minutes, followed by a maintenance dose of 0.54 mg/kg/h, given as an infusion over 60 min. Dialysate, i.e., probe perfused Ringer’s solution, containing the internal standard oxycodone-D3, was sampled in 10-min intervals and blood was sampled at indicated time points. The brain was isolated and dissected at the end of the experiment. Dialysate from CNS and blood probes, plasma, blood cells (BC) and brain tissue (whole brain, right- and left striatum) samples were analyzed by UPLC-MS/MS for quantification of analytes. Selected brain striatal area and plasma samples were analyzed using the Olink Target 96 Mouse Exploratory assay for identification and quantification of various biomarkers. Oxycodone PK data analysis was performed using Phoenix 64 WinNonLin. **B** Illustration of brain sampling sites: R STR, right striatal ipsilateral area of microdialysis probe placement; L STR, left contralateral site, and WB, remaining right hemisphere after sampling of the striatal area, named whole brain

An overview of the experimental procedure is presented in Fig. 1A. Surgery was performed as previously described [2]. Two of the following CNS probes were placed in each rat: a 3 mm CMA 12 Elite probe (CMA Microdialysis AB, Kista, Sweden) for striatum, 1 mm CMA 12 Elite probes (CMA Microdialysis AB, Kista, Sweden) for the lateral ventricle and *cisterna magna*. Pending the experiment, the rat was housed individually in a CMA/120 system for freely moving animals (CMA Microdialysis AB, Kista, Sweden), with food and water ad libitum for approximately 27 h to allow for recovery. The surgeries were performed between 7–10 a.m. to enable start of the experiment at similar time points as our previous study in healthy rats [2]. Surgical instruments were sterilized using a glass bead dry sterilizer (Simon Keller AG, Switzerland) at 230–250 °C for 15 s and surgeries were performed at aseptic conditions.

### Lipopolysaccharide administration

LPS challenge was selected as an inflammation model. Ten rats of both sexes (n_female_ = 4, n_male_ = 6) were administered LPS from *Salmonella typhimurium* intraperitoneally (ip) at three doses of 3 mg/kg starting at the time of microdialysis surgery, and at 6 and 24 h thereafter, according to previously published protocols in mice [26–28]. This regimen previously resulted in elevated cytokines, such as interleukin-6 (IL-6), interleukin-1α (IL-1α), and interleukin-1β (IL-1β) in serum as well as cortex and hippocampus in mice [26, 27], confirming achievement of systemic and central immune responses.

LPS was dissolved in sterile saline at a concentration of 2 mg/mL. The ip injection volume of each dose was around 500 µL, depending on the rat's body weight. The LPS solution was stored in polypropylene SafeSeal micro tubes (Sarstedt AG & Co. KG, Nümbrecht, Germany) at −80 °C according to the manufacturer’s recommendations. After the LPS solution was thawed, it was mixed using a magnet stirrer for 15 min, and at the time of the experiment, the LPS solution was vortexed to avoid injecting a sedimented solution. The total dose of LPS administered in the three doses was 9 mg/kg. The first oxycodone infusion (Dosing regimen I) was initiated 28 h after the first LPS injection.

### Microdialysis experiment

The experimental design, including animal handling, oxycodone dosing, microdialysis experiment, and bioanalysis was the same as that reported in Bällgren et al. [2]. Briefly, the experiment consisted of (i) a 60-min stabilization period, (ii) a 60-min constant-rate infusion (Dosing regimen I), (iii) a 180-min post-infusion period, and (iv) a 60-min steady-state period (Dosing regimen II, Fig. 1A). The stabilization period implied probe perfusion with Ringer’s solution containing 44 ng/mL of the calibrator oxycodone-D3, for assessment of probe recovery [29]. Throughout the experiment, the microdialysis probes were perfused with a filtered Ringer’s solution (pH 7.4) containing the calibrator at a flow rate of 1 µL/min, using a CMA 400 Syringe Pump (CMA Microdialysis AB, Kista, Sweden). The oxycodone infusions were performed with a Harvard 22 pump (Harvard Apparatus Inc., Holliston, MA). All ten rats received a 60-min constant-rate infusion of 0.3 mg/kg/h oxycodone (Dosing regimen I), followed by a washout period (post-infusion period) and a 60-min steady-state condition induced by a loading dose of 0.24 mg/kg and a maintenance dose 0.54 mg/kg/h (Dosing regimen II). Dosing regimen I enables analysis of both the rate and extent of oxycodone transport across the CNS barriers and Dosing regimen II provides a steady-state condition required for investigation of CNS drug delivery as well as plasma protein binding and brain tissue binding/uptake.

Dialysate samples were collected in pre-weighed vials (polypropylene with polyurethane caps; AgnTho’s, Lidingö, Sweden) in 10-min intervals, and, thereafter, weighed, capped, and stored at 6 °C. Blood was sampled in heparinized (5 µL 5000 IU/mL heparin) Eppendorf tubes (polypropylene; Eppendorf, Hamburg, Germany) before the first LPS dose and at 5, 15, 25, 45, 55, 75, 85, 115, 175 and 275 min after initiation of Dosing regimen I. A maximum of 180 µL per blood sample and minimum 10 mL of terminal blood were taken from each rat. The blood samples were centrifuged (MicroStar12 centrifuge; VWR International AB, Stockholm, Sweden; or IEC Centra CL2, Labora; IEC international equipment company, Needham Heights, MA, USA) and plasma was transferred to new Eppendorf tubes and stored at −20 °C. After the terminal blood sampling, the brain was isolated and dissected. Striatum from both hemispheres, and the remaining tissue from one hemisphere, were collected; the right striatum as a reference for probe placement, and the left as a reference without probe. The brains were visually examined for confirmation of correct probe placements and notations of possible macro changes related to the implantation of the probes. The brain samples were stored covered by aluminum foil in polystyrene cups (VWR International, Radnor, USA) at −80 °C.

### In vivo recovery calculation

To correct for incomplete recovery across the probe membranes and enable estimation of unbound oxycodone concentrations at probe sites, retrodialysis by calibrator was performed in vivo throughout the experiment [29]. The method assumes that the probe recovery loss of the calibrator, here oxycodone-D3, is equal to the gain recovery of the drug, which is a valid assumption as oxycodone and oxycodone-D3 are almost identical molecules. The probe recovery was calculated as follows:

1 $$Recovery = {\raise0.7ex\hbox{${\left( {C_{in} - C_{out} } \right)}$} \!\mathord{\left/ {\vphantom {{\left( {C_{in} - C_{out} } \right)} {C_{in} }}}\right.\kern-0pt} \!\lower0.7ex\hbox{${C_{in} }$}}$$

where C_in_ is the mean calibrator concentration in the perfusion solution entering the probe, sampled from the perfusion syringes before and after the experiment. C_out_ is the mean concentration of the calibrator in the dialysate samples leaving the probe, collected from each probe throughout the experiment. Mean recoveries were calculated for each probe membrane length, i.e., the blood probe [10 mm], striatum probe (3 mm) and the lateral ventricle and *cisterna magna* probes (1 mm), as the recovery is dependent on the membrane length. As the recovery from probes with shorter membranes is more sensitive to fluctuations, mean recoveries were used for the estimations of unbound oxycodone concentrations in striatum, lateral ventricle, and *cisterna magna*. To exclude unrealistic recovery values and determine which values to include in the mean estimations, previously published oxycodone recoveries were used as references [1, 30]. Recoveries within two standard deviations (SD) around the mean of the previously published values were included.

### Measurement of oxycodone partitioning into blood cells after LPS challenge

The blood-to-plasma concentration ratio (C_b_/C_p_) of oxycodone has been reported to be above unity [1, 2]. The status of oxycodone red blood cell partitioning as well as hematocrit were therefore investigated in the LPS-treated rats (n = 6), as by the previous protocol [2]. The C_b_/C_p_ ratio was calculated as follows, according to Tozer [31]:

2 $${\raise0.7ex\hbox{${C_{b} }$} \!\mathord{\left/ {\vphantom {{C_{b} } {C_{p} }}}\right.\kern-0pt} \!\lower0.7ex\hbox{${C_{p} }$}} = 1 - Hct + Hct \times \left( {{\raise0.7ex\hbox{${C_{BC} }$} \!\mathord{\left/ {\vphantom {{C_{BC} } {C_{plasma} }}}\right.\kern-0pt} \!\lower0.7ex\hbox{${C_{plasma} }$}}} \right)$$

where C_BC_, and C_plasma_ are total concentrations in blood cells (BC), and plasma, respectively, and Hct is the hematocrit.

### Bioanalysis

The bioanalysis of dialysate, plasma, blood cells, and brain samples as well as the respective standards, quality controls (QCs), and blanks, was performed according to the previously described protocol [2].

### Proteomic analysis

To confirm the achievement of inflammation induced by LPS, proteomic analysis was performed on randomly selected animals using the Target 96 Mouse Exploratory panel (Olink Proteomics AB, Uppsala, Sweden) which includes quantification of well-known inflammation biomarkers (Table S1). The analysis is based on Proximity Extension Assay (PEA) technology, where oligonucleotide-labeled antibody probe pairs bind to their respective target protein present in the sample. According to the manufacturer, the Target 96 Mouse Exploratory panel can be extended to analyze rat proteins.

**Fig. 2:**
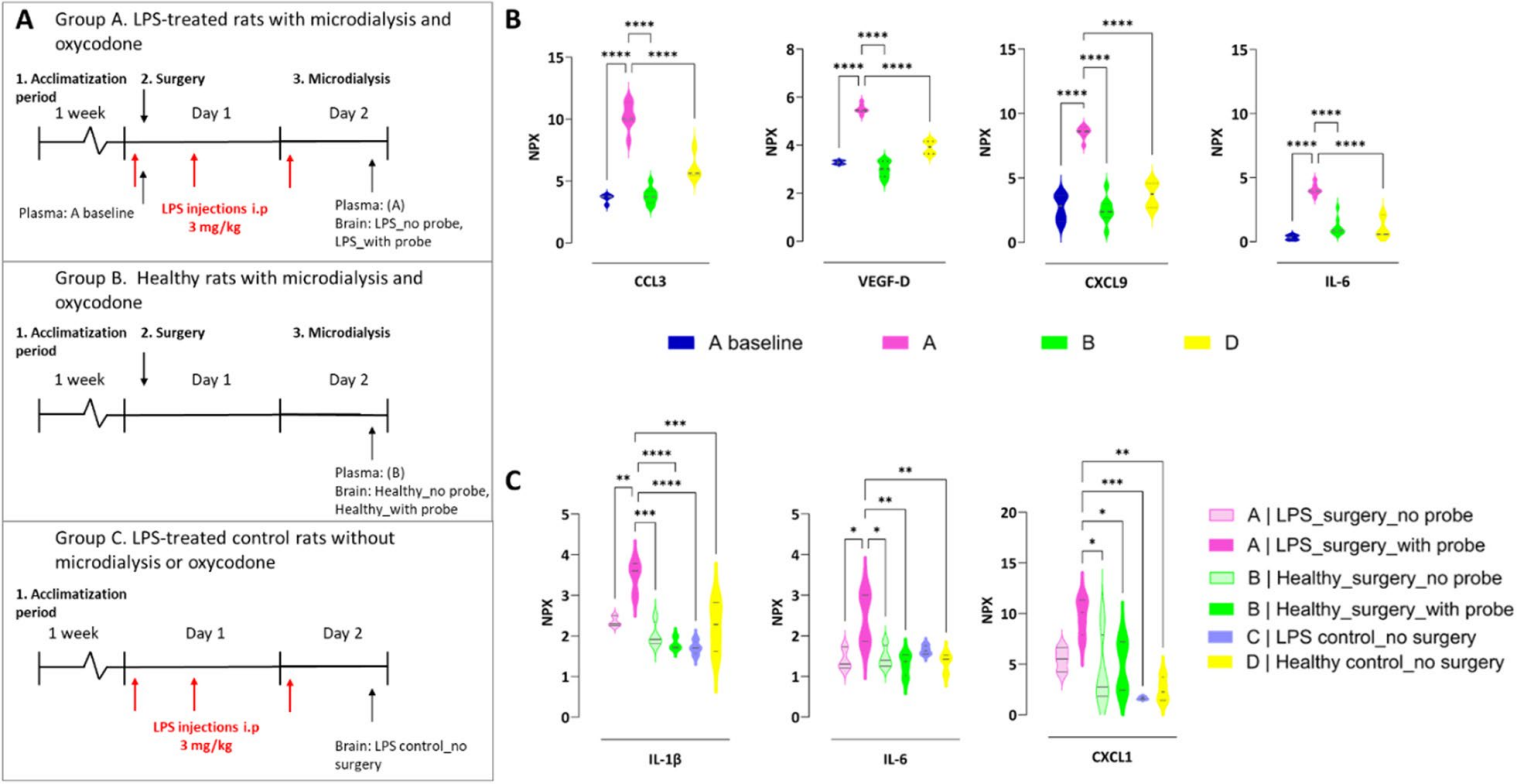
LPS model characterization by Olink Target 96 Mouse Exploratory assay. **A** A schematic overview of samples collected for biomarker analysis in Group **A**) LPS-treated rats with microdialysis and oxycodone, Group **B**) healthy rats with microdialysis and oxycodone, Group **C**) LPS-treated control rats without microdialysis or oxycodone, and Group D) healthy controls without microdialysis, LPS treatment or oxycodone administration (not indicated in the figure). **B** Violin plots of normalized protein expression (NPX) median values and quartiles of proteins. The most pronounced changes found in plasma are C–C motif chemokine ligand 3 (CCL3), vascular endothelial growth factor D (VEGF-D), C-X-C motif chemokine ligand 9 (CXCL9), and interleukin-6 (IL-6). **C** Violin plots of NPX of three proteins with the significant changes in the brain, i.e., interleukin-1β (IL-1β), interleukin-6 (IL-6), and C-X-C motif chemokine ligand 1 (CXCL1). Comparisons of protein levels in plasma and brain between the groups were performed using ANOVA followed by post-hoc analysis using Tukey’s tests. *p < 0.05, **p < 0.01, ***p < 0.001, ****p < 0.0001. Detailed information on statistical analysis of all proteins is presented in Supplementary materials, Excel File: Proteomics_Plasma_Brain.xlsx

The proteins were quantified in plasma and brain, i.e., right striatum (ipsilateral side with brain probe placement) and left striatum (contralateral side with no probe placement). An overview of the four study groups (n = 4 per group), and the collection of plasma and brain samples used for the proteomic analysis, is presented in Fig. 2A. Briefly, samples from the following groups were included: A. LPS-treated rats after microdialysis and oxycodone, B. Healthy rats after microdialysis and oxycodone, C. LPS-treated controls without microdialysis or oxycodone, and D. Healthy controls without any intervention (microdialysis, LPS challenge or oxycodone). All groups included an even distribution of females and males. Rats in the A and B groups had undergone all steps of the microdialysis study (Fig. 1A) with or without the LPS challenge, respectively. Rats in the C and D groups only went through acclimatization without microdialysis or oxycodone, and with or without LPS challenge, respectively. In group C, terminal samples were collected approximately eight hours after the third LPS dose, to match the terminal sampling time point of the rats included in the microdialysis study group A. Both plasma samples and brain homogenate samples were randomly organized on 96-well plates, and pseudonymized sample identification numbers were used. Samples were analyzed at Olink Proteomics AB Lab in Uppsala, Sweden. Data were reported using a Normalized Protein Expression (NPX) unit. NPX is on a log2 scale, i.e., a larger number represents a higher protein level in the sample, typically with a background level around zero.

### Assessment of BBB integrity

The integrity of the BBB was assessed in a separate group of LPS-treated (n = 4) and healthy (n = 3) Sprague–Dawley male rats by evaluating the extent of a fluorescent 4 kDa tetramethyl-rhodamine B isothiocyanate (TRIC) dextran transport across the barrier as previously described [32, 33]. Rats underwent surgery one day before the experiment. The surgery included femoral vein and artery catheterization for dextran administration and blood sampling, respectively. In addition, the placement of a microdialysis probe guide into the right striatum of all animals was performed, aiming to mimic the experimental conditions during the microdialysis studies. LPS was administered as described above. On the day of experiment, a 5-min iv infusion of dextran was administered at a dose of 400 mg/kg using a Harvard 22 pump (Harvard Apparatus Inc., Holliston, MA). Blood was sampled before the start of the infusion and 5 min after the start of the infusion. Terminally at 5 min, blood was sampled by heart puncture, followed by transcardial perfusion using 0.9% NaCl at a rate of 10 mL/min for 2 min. The blood samples were allowed to coagulate for 15–20 min at room temperature before it was centrifuged at 10,000 rpm for 5 min. Thereafter, serum was collected, diluted 1:9 (v:v) in MilliQ water, and stored at 6 °C until bioanalysis. The brain was isolated and dissected into the two hemispheres, followed by microdissection of the left and right striatum (Fig. 1B). Left and right striatal areas, and the remaining tissue of the right hemisphere (referred to as the whole brain, WB) were weighed and homogenized 1:3 (w:v) in MilliQ water using a 4-Place Beads Homogenizer (VWR, Stockholm, Sweden). Samples were centrifuged at 14800 rpm for 1 h at 4 °C, followed by supernatant collection. Tecan Spark^®^ Multimode Microplate Reader (Tecan Group Ltd., Männedorf, Switzerland) was used to measure the intensity of the fluorescent TRITC dextran at an excitation wavelength of 550 nm and an emission wavelength of 571 nm. The highest standard was used to optimize the signal. Brain-to-serum concentration ratio was estimated using the terminal blood and brain samples.

### Data analysis for PK parameters

For the estimation of unbound oxycodone concentrations at the sites of probe placement, the concentrations measured in the dialysate samples were divided by recovery of the probe, according to the following equation:

3 $$C_{u} = {\raise0.7ex\hbox{${C_{dialysate} }$} \!\mathord{\left/ {\vphantom {{C_{dialysate} } {Recovery}}}\right.\kern-0pt} \!\lower0.7ex\hbox{${Recovery}$}}$$

where C_u_ is the unbound concentration at the probe location, C_dialysate_ is the concentration in the dialysate sample, and Recovery (Eq. 1) is the calibrator recovery across the probe membrane. To estimate unbound concentrations in blood, individual recoveries were used, and to estimate unbound concentrations in the striatum, lateral ventricle, and *cisterna magna*, mean recoveries for each probe membrane length were used.

For estimation of total oxycodone concentrations in blood (C_blood_) from the measured total concentrations in plasma (C_plasma_), the total concentration in plasma was multiplied with C_b_/C_p_, according to the following equation:

4 $$C_{blood} = C_{plasma} \times {\raise0.7ex\hbox{${C_{b} }$} \!\mathord{\left/ {\vphantom {{C_{b} } {C_{p} }}}\right.\kern-0pt} \!\lower0.7ex\hbox{${C_{p} }$}}$$

PK parameters, including the area under the concentration–time curve extrapolated to infinity (AUC_inf_obs_), and normalized by oxycodone dose (AUC_inf_D_obs_), and from time 0–180 min (AUC_last_), clearance (CL__obs_), the volume of distributions (V_z_obs_ and V_ss_obs_) and terminal half-life (t_1/2_) were estimated from data obtained from Dosing regimen I using Phoenix 64 WinNonLin (Certara, New Jersey, USA) as previously described [2]. The total and unbound concentrations at steady-state (C_ss_ and C_u,ss_) were calculated as the mean concentration of samples collected 35 min after initiation of the loading dose of Dosing regimen II.

The fraction of unbound drug in the blood (f_u,blood_) was calculated as follows:

5 $$f_{u,blood} = {\raise0.7ex\hbox{${C_{u,blood,ss} }$} \!\mathord{\left/ {\vphantom {{C_{u,blood,ss} } {C_{tot,blood,ss} }}}\right.\kern-0pt} \!\lower0.7ex\hbox{${C_{tot,blood,ss} }$}}$$

where C_u,blood,ss_ is the mean unbound concentration in blood at steady-state and C_tot,blood,ss_ is the mean total concentration in blood at steady-state.

The unbound partition coefficients, describing the extent of drug delivery to striatum (K_p,uu,STR_), lateral ventricle (K_p,uu,LV_) and *cisterna magna* (K_p,uu,CM_) were estimated as follows:

6 $$K_{p,uu} = {\raise0.7ex\hbox{${AUC_{u,brain} }$} \!\mathord{\left/ {\vphantom {{AUC_{u,brain} } {AUC_{u, blood} }}}\right.\kern-0pt} \!\lower0.7ex\hbox{${AUC_{u, blood} }$}}$$

and

7 $$K_{p,uu} = {\raise0.7ex\hbox{${C_{u,brain,ss} }$} \!\mathord{\left/ {\vphantom {{C_{u,brain,ss} } {C_{u,blood,ss} }}}\right.\kern-0pt} \!\lower0.7ex\hbox{${C_{u,blood,ss} }$}}$$

To compare the extent of drug delivery between two CNS sites, the relative extent was determined based on the ratio of the respective K_p,uu_ values:

8 $$Relative\ extent_{CNS1/CNS2} = {\raise0.7ex\hbox{${K_{p,uu,CNS1} }$} \!\mathord{\left/ {\vphantom {{K_{p,uu,CNS1} } {K_{p,uu,CNS2} }}}\right.\kern-0pt} \!\lower0.7ex\hbox{${K_{p,uu,CNS2} }$}}$$

The partition coefficient between total brain and blood concentrations, K_p,brain_, was estimated as follows:

9 $$K_{p,brain} = {\raise0.7ex\hbox{${C_{tot,brain,ss} }$} \!\mathord{\left/ {\vphantom {{C_{tot,brain,ss} } {C_{tot, blood,ss} }}}\right.\kern-0pt} \!\lower0.7ex\hbox{${C_{tot, blood,ss} }$}}$$

where C_tot,brain,ss_ is the total concentration in the brain (right and left striatum, and whole brain) at steady-state, and C_tot,blood,ss_ is the total concentration in blood at steady-state.

The apparent unbound volume of distribution in the brain (V_u,brain_), describing the intra-brain distribution, was calculated as follows:

10 $$V_{u,brain} = {\raise0.7ex\hbox{${A_{brain} }$} \!\mathord{\left/ {\vphantom {{A_{brain} } {C_{u,brainISF} }}}\right.\kern-0pt} \!\lower0.7ex\hbox{${C_{u,brainISF} }$}}$$

where A_brain_ is the amount of drug in the right striatum corrected for the residual blood in the brain and C_u,brainISF_ is the unbound concentration in brain ISF.

### Statistical analysis

GraphPad Prism version 9.0.0 for Windows (GraphPad Software, San Diego, California USA, http://www.graphpad.com) was used for statistical analyses. All data are presented as mean ± SD. Shapiro–Wilk normality test was used to test normal distribution, and if passed, parametric statistical tests were used for data analysis. Significant differences were indicated by p < 0.05. Principal component analysis (PCA) was performed on plasma and brain proteomic datasets using SIMCA 17 (Sartorius, Sweden).

## Results

### Elevated inflammation biomarkers in plasma and brain after LPS challenge

To characterize the LPS model, animals from the four A-D groups were included in the biomarker analysis, to evaluate potential changes in protein levels during microdialysis, LPS challenge, and their combination, as well as baseline levels in healthy animals (Fig. 2A). In plasma, 79 out of 92 biomarkers showed significant differences between the groups (Supplementary Materials: Proteomics_plasma_brain.xlsx). The most pronounced changes in plasma were observed for C–C motif chemokine ligand 3 (CCL3), vascular endothelial growth factor D (VEGF-D), C-X-C motif chemokine ligand 9 (CXCL9), and interleukin-6 (IL-6) (Fig. 2B). The levels of these biomarkers were significantly higher in plasma from LPS-treated rats in the microdialysis oxycodone study (Group A) compared to the healthy group (Group B). This confirms that cytokine and biomarker levels were significantly elevated in LPS-treated rats with microdialysis and oxycodone treatment. Additionally, there was a significant increase in these biomarkers in the LPS-treated rats between plasma sampled after one dose of LPS (Group A: A baseline) and plasma sampled after three doses of LPS (Group A), confirming the dose- and time-dependent changes in immune response. In contrast to plasma, only three out of 92 proteins, interleukin-1β (IL-1β), IL-6, and C-X-C motif chemokine ligand 1 (CXCL1), were significantly different between the groups in the brain samples. These proteins showed marked elevations at the site of probe placement in LPS-treated rats included in the microdialysis oxycodone study (A) (Fig. 2C).

**Fig. 3:**
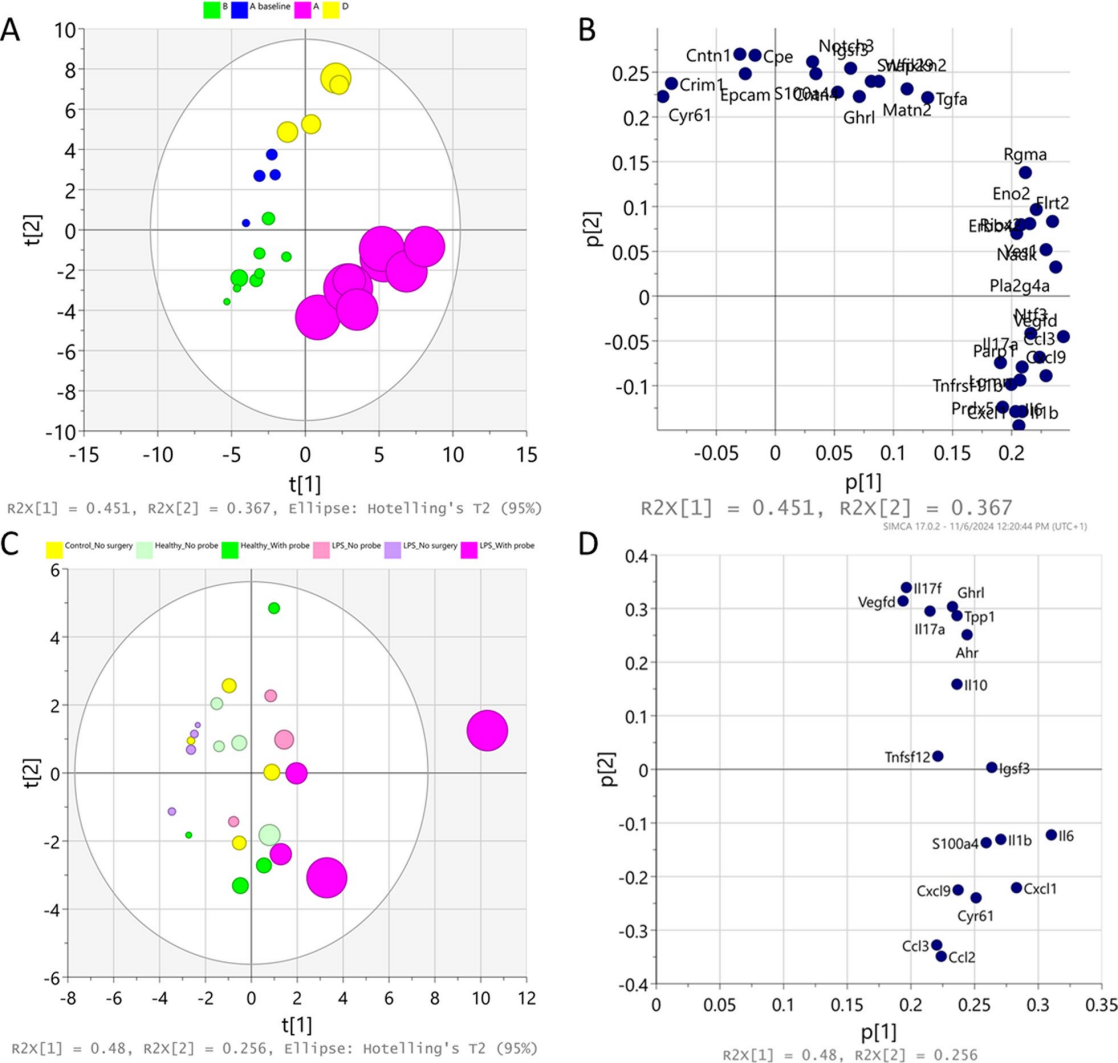
Principal component analysis (PCA) reveals a distinct separation between investigated groups based on plasma proteome, but not the brain proteome. PCA scores and loading plots of the first two principal components of a data set of proteomic analysis obtained from plasma (**A**, **B**) and brain striatal area (**C**, **D**) of LPS-treated and healthy rats. **A** Plasma samples are colored by groups: Group A: rats in the microdialysis experiment with full LPS challenge, i.e., three doses of LPS (purple); Group **A** baseline: rats with microdialysis surgery and only one dose of LPS (blue); Group **B**: rats in the microdialysis experiment without LPS challenge (green); and Group **D**: rats without any microdialysis surgery or LPS challenge (yellow). The size of the circles reflects abundance of C–C motif chemokine ligand 3 (CCL3) in the samples, the bigger circle the larger abundance. **C** Brain samples are colored by the groups: Group **A**: striatal areas from the rats in the microdialysis experiment with full LPS challenge at the ipsilateral probe site (purple) and its contralateral site (light purple); Group **B**: striatal areas from the rats in the microdialysis experiment without LPS challenge at the probe site (green) and its contralateral site (light green); Group **C**: brains from rats receiving only LPS challenge (violet), and Group **D**: brain from rats without any microdialysis surgery, LPS challenge or oxycodone administration (yellow). The size of the circles reflects abundance of interleukin-6, the bigger the larger. The differential biomarker profiles between plasma and brain highlight the compartmentalized nature of the inflammatory response. While systemic inflammation was robustly evident, the brain’s response appeared more localized and restricted. This raises important questions about the origin of brain cytokines. An expanded version of the plasma loading plot is presented in the Supplementary material (Fig. S1)

The PCA on proteomic data in plasma revealed a distinct separation between all investigated groups (Fig. 3A). The two principal components accounted for 45% and 37% of the variation seen in the plasma profiles of investigated groups. The loading plot highlighted the markers with the most significant impact on each component, with CCL3, VEGF-D, contactin 1 (CNTN1), and IL-6, showing an intermediate influence on both components (Fig. 3B). In contrast, the PCA on proteomics data from the brain did not show a clear separation between groups (Fig. 3C). Three principal components explained 75% of the variability in the brain observed between groups. The loading plot revealed IL-6, C–C motif chemokine ligand 2 (CCL2), CCL3, VEGF-D, and interleukin 17f and 17a (IL17f, IL17a) having an intermediate influence on the first two components (Fig. 3D).

The scrutiny of biomarkers confirms achievement of a stable immune response in blood and brain, indicating systemic inflammatory and, possibly, neuroinflammatory effects of the LPS treatment. However, the origin of brain cytokines remains uncertain, raising questions about whether they are produced locally in the brain or transported from the peripheral circulation across the brain barriers.

### Impaired blood–brain barrier integrity after LPS challenge

**Fig. 4:**
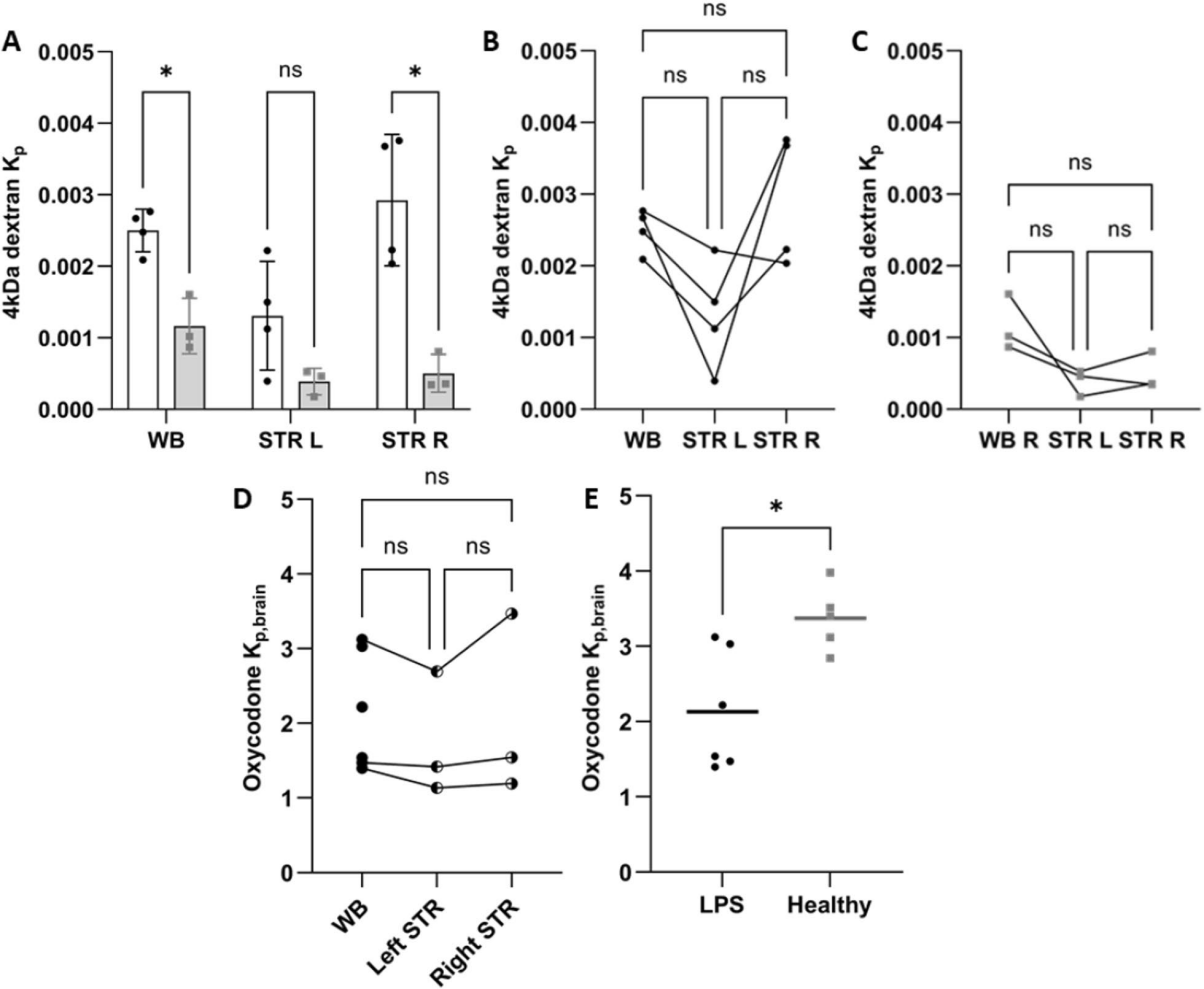
Opposing impact of LPS challenge on paracellular transport at the blood–brain barrier measured by 4 kDa TRITC dextran and oxycodone total brain-to-plasma concentration ratio, K_p,brain_. **A** Scatter dot plots of the total 4 kDa TRITC dextran brain-to-serum concentration ratios reflecting the extent of paracellular transport at the BBB in LPS-treated (black dots, open bars) and healthy (gray squares and bars) rats. The top of the bars indicates the mean and the whiskers describe the SD. **B** and **C**) Intra-subject variability in the site-specific paracellular transport measured in LPS-treated rats (**B**) and healthy controls (**C**). Data and statistical test results of the 4 kDa dextran are presented in Table S3. **D**) Scatter dot plots of the total oxycodone brain-to-plasma concentration ratios (K_p,brain_) in WB, STR L, and STR R in LPS-treated rats. Data are presented as individual paired data points. **E** Scatter dot plots of the mean oxycodone K_p,brain_ measured in LPS-treated (black dots) and healthy (gray squares) rats. An unpaired two-tailed t-test was used to compare oxycodone K_p,brain_ between LPS-treated and healthy rats (p = 0.01). *p < 0.05. ns = not significant. NB: Please note that the lines in graphs C, D, and E are merely used to connect the samples from the same subject

To evaluate BBB integrity, we selected 4 kDa TRITC dextran as a marker of paracellular transport. In the LPS-treated rats, the brain-to-serum concentration ratio for 4 kDa TRITC dextran was 0.0013 (or 0.13%) in the left striatal area, 0.0029 (0.29%) in the right striatal area, and 0.0025 (0.25%) in the whole brain (Fig. 4A-B, Table S2). These ratios were, on average, higher than those in healthy rats with a dramatic 5.8-fold increase (p = 0.03) observed in the right striatal area with probe placement (Fig. 4A, Table S3). The ratios had considerable inter-subject variability, with a mean coefficient of variation of 30% across all groups. There were no regional differences in the paracellular transport between right and left striatum and whole brain, reflected by the dextran K_p_ ratios, in either LPS-treated (p = 0.11) or healthy rats (p = 0.14) (Fig. 3C, D).

The study with the paracellular marker 4 kDa TRITC dextran confirmed that BBB integrity was compromised following the LPS challenge. The BBB exhibited more pronounced “leakiness” in the right striatal area with probe placement after LPS administration, yet the extent of paracellular transport did not exceed 0.29%.

### Reduced extent of oxycodone uptake across the blood–brain barrier following LPS challenge

**Fig. 5:**
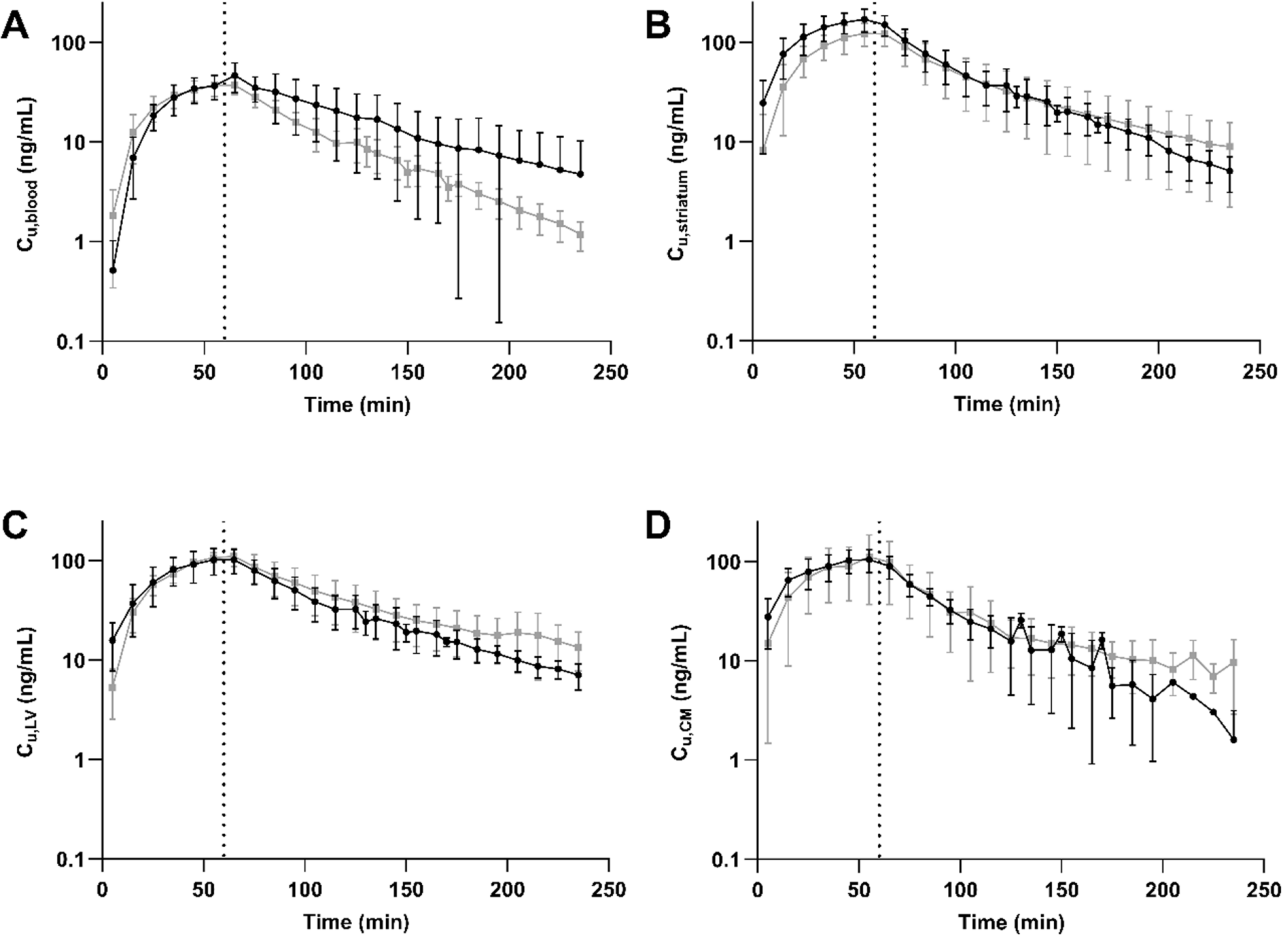
Unbound oxycodone concentration–time profiles in blood, striatum, lateral ventricle (LV), and *cisterna magna* (CM) in LPS-treated and healthy rats. Mean unbound oxycodone concentration–time profiles in LPS-treated (black dots) and healthy (gray squares) rats in **A**) blood (n_healthy_ = 16, n_LPS_ = 6), **B** STR (n_healthy_ = 16, n_LPS_ = 6), **C** LV (n_healthy_ = 9, n_LPS_ = 3), and **D** CM (n_healthy_ = 7, n_LPS_ = 3). Data is presented as mean ± SD

**Fig. 6:**
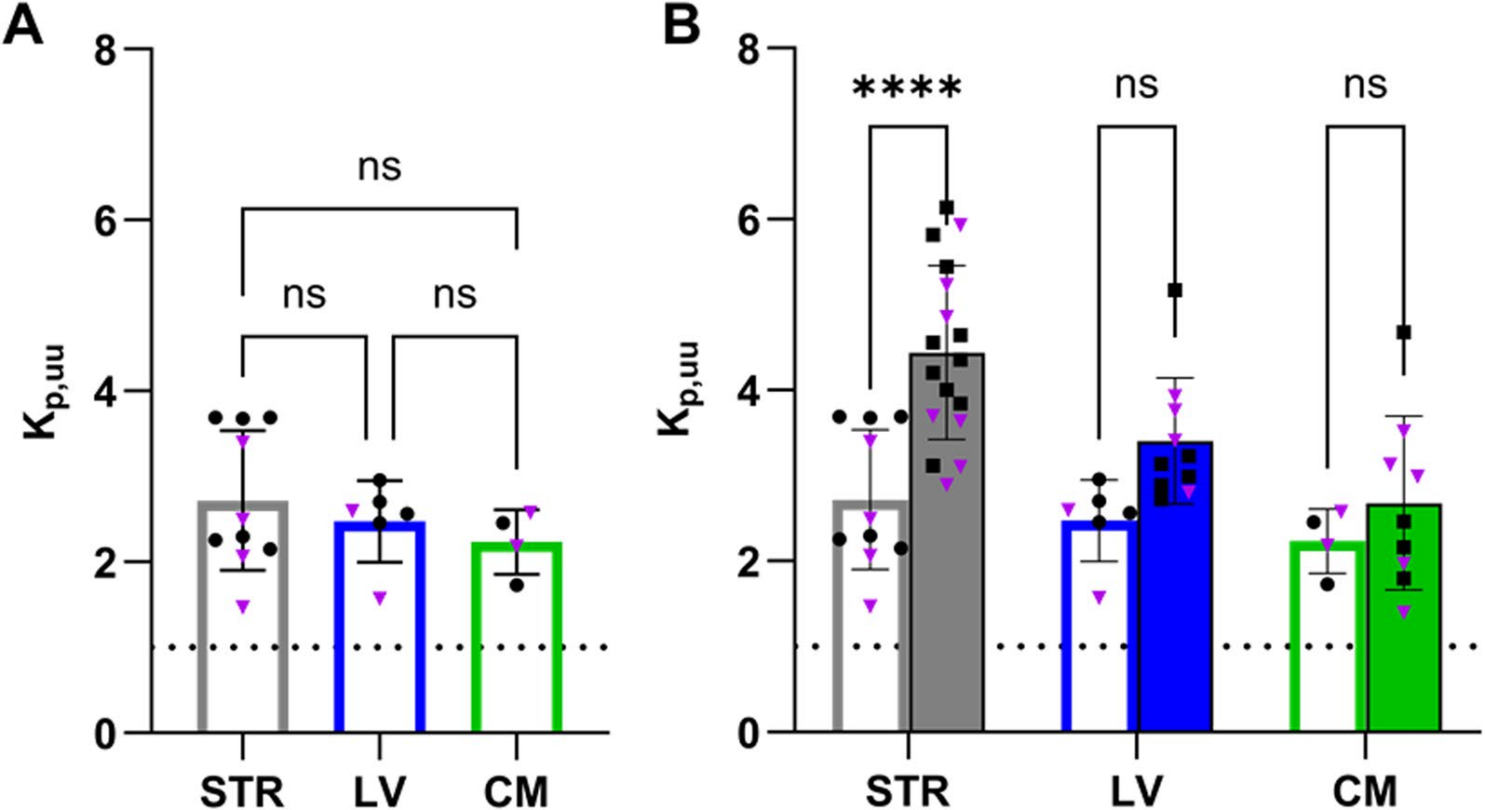
Scatter dot plots of K_p,uu_ estimates of striatum (STR, gray), lateral ventricle (LV, blue), and cisterna magna (CM, green). **A** LPS-treated rats (open bars). **B** Comparison of K_p,uu_ estimates obtained in LPS-treated (open bars) and healthy rats (filled bars). Healthy data are modified from Bällgren et al. [2]. The means are indicated by the top of the bar and whiskers represent the SD. Female subjects are indicated by purple triangles and males by black circles and squares. Data and statistical test details are presented in Tables 1, S4, S5. ****p < 0.0001, ns = not significant

To characterize unbound oxycodone transport across the BBB after LPS treatment, we used brain microdialysis. The unbound concentration–time profile of oxycodone in the different CNS sites was similar between LPS-treated and healthy rats (Fig. 5B-D), with no significant differences in the mean exposure (AUC_inf_D_obs_) in striatum, lateral ventricle, or *cisterna magna* (p = 0.89). The mean K_p,uu,STR_ value, describing the extent of oxycodone transport across the BBB measured in striatum after the LPS challenge, was 2.72, indicating that active uptake into the brain was still present despite the challenge with LPS (Fig. 6, Table 1, Table S4, S5). However, this represents approximately 60% of the oxycodone uptake previously reported in healthy rats, i.e., K_p,uu,STR_ of 2.72 vs 4.4 in LPS-treated and healthy rats, respectively (p < 0.0001) [2]. There were no differences in K_p,uu_ obtained based on AUC or Css; Eq. 7–8, Table 1, something that was also expected. No sex differences were observed in the extent of BBB transport either in LPS-treated or healthy groups, measured by an ordinary two-way ANOVA, (p = 0.20, Table 1, Table S5). However, there was a non-significant trend (p = 0.28) of higher striatal ISF exposure in females, with mean AUC_last_D_ 0–235-min values of 44880 vs. 34,390 min·ng·mL^−1^/(mg/kg) in females and males, respectively (Fig. S2). These were also similar as the striatal exposure in healthy female and male rats with AUC_last_D_ of 40200 and 41440 min·ng·mL^−1^/(mg/kg), respectively. Additionally, measuring total brain-to-plasma concentration ratios, revealed lower K_p,brain_ values in LPS-treated rats compared to healthy rats, with K_p,brain_ of 2.13 ± 0.79 (N = 6) and 3.4 [2] in LPS-treated and healthy rats, respectively (p = 0.01, Fig. 4D-E).

**Table 1:** K_p,uu_ in striatum (STR), lateral ventricle (LV), and *cisterna magna* (CM) estimated using Dosing regimen I and Dosing regimen II, in LPS-treated rats

	Dosing regimen I			Dosing regimen II
	*Both sexes*	*Females*	*Males*	*Both sexes*
K_p,uu,STR_	2.72 ± 0.82 (n = 10)	2.36 ± 0.81 (n = 4)	2.96 ± 0.80 (n = 6)	2.97 ± 0.84 (n = 7)
K_p,uu,LV_	2.47 ± 0.47 (n = 6)			2.59 ± 0.66 (n = 4)
K_p,uu,CM_	2.24 ± 0.38 (n = 4)			2.16 ± 0.81 (n = 3)

K_p,uu_ estimations using Eq. 7 for Dosing regimen I, Eq. 8 for Dosing regimen II

Mean ± SD. There were no significant differences between K_p,uu_ of STR, LV and CM. Compared to healthy, K_p,uu,STR_ was lower (p < 0.0001), while K_p,uu,LV_ (p = 0.12) and K_p,uu,CM_ (p = 0.78) were similar after LPS treatment. There were no differences in K_p,uu,STR_ between females and males (p = 0.92). Further statistical test details are presented in Tables S4, S5

The unbound oxycodone brain-to-plasma concentration ratios at single time points in striatum and lateral ventricle (C_u,STR_/C_u,blood_ and C_u,LV_/C_u,blood_) were higher at early time points post-oxycodone administration (Fig. S3A and C). These ratios stabilized within 30 min and were concentration independent (Fig. S3).

The assessment of oxycodone delivery across the BBB revealed a significant reduction in active uptake following LPS administration. Nonetheless, the mean exposure in the striatal ISF was not significantly different compared to healthy rats.

### No difference in the oxycodone exposure between striatal interstitial fluid and ventricular or cisternal cerebrospinal fluids

To investigate the extent of oxycodone transport across the BCSFB as well as CSF-ISF exchange after LPS administration, we dynamically monitored unbound oxycodone concentrations in the lateral ventricle and *cisterna magna*. Although a reduced extent of oxycodone uptake was observed in brain ISF, this reduction did not reach statistical significance in the CSF compartments, as indicated by similar K_p,uu,LV_ (p = 0.12) and K_p,uu,CM_ (p = 0.78) after LPS treatment. The relative extent of oxycodone delivery to striatum compared to that in the lateral ventricle was 1.11 ± 0.26 (range: 0.78–1.44; n = 6), and to *cisterna magna* was 1.24 ± 0.52 (range: 0.92–2.13; n = 4), with no significant differences. A mixed-effects analysis on K_p,uu_ values in the LPS-treated rats, revealed similar extent of distribution into striatum, lateral ventricle, and *cisterna magna* (p > 0.05, Table 1, Fig. 6). This suggests there is no difference in the extent of oxycodone transport across the BBB and the BCSFB after the LPS challenge. Yet, in healthy rats, the extent of transport across the BBB was previously reported to be higher than that across the BCSFB [2].

### Sex differences in systemic pharmacokinetics of oxycodone after LPS challenge

**Fig. 7:**
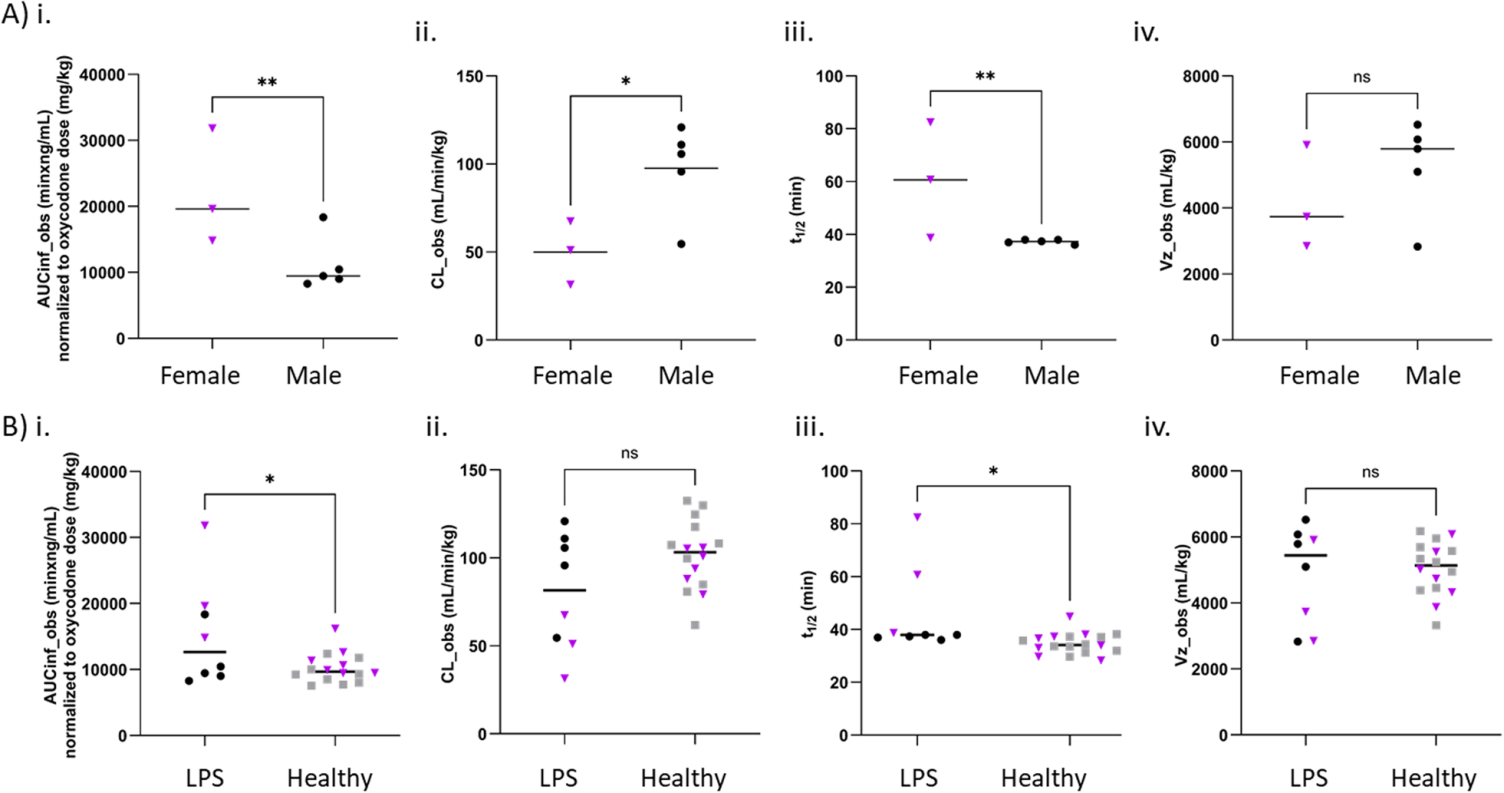
Alterations in systemic pharmacokinetics of oxycodone in LPS-treated female rats. Scatter dot plots comparing unbound oxycodone blood pharmacokinetic parameter estimates: (i) area under the concentration–time curve (AUC_inf_obs_) normalized by oxycodone dose, (ii) clearance (CL__obs_), (iii) half-life (t_1/2_), and (iv) volume of distribution based on the terminal phase (V_z_obs_). **A** between LPS-treated male and female rats, and **B** between LPS-treated and healthy rats. In **B**, female subjects are indicated by purple triangles and males are indicated by black dots or grey squares. Comparisons were performed using unpaired two-tailed t-tests; *p < 0.05, **p < 0.01

To describe the systemic PK of oxycodone, we longitudinally measured unbound and total oxycodone concentrations in blood and plasma. We observed a significant difference in the exposure of unbound oxycodone in blood (AUC_inf_D_obs_) between LPS-treated and healthy rats, driven by the differences between LPS-treated and healthy female rats (Fig. 7, Table S6). The systemic exposure of unbound oxycodone in LPS-treated female rats was significantly two-fold higher than in healthy females (p = 0.002), while the difference among males was insignificant (Fig. 7, Table S7). Thus, AUC_inf_D_obs_ of unbound oxycodone in blood was higher in LPS-treated females than in males (Fig. 7, Tables S6, S7). This was also reflected in total oxycodone blood concentrations (Fig. S4). These differences in systemic exposure were due to differences in clearance (CL__obs_), where LPS-treated female rats exhibited lower clearance than healthy females, resulting in a 1.7-time longer half-life of oxycodone, i.e., 61 min, compared to both LPS-treated males and healthy females (Fig. 7, Tables S6, S8, S9). The estimated volumes of distribution, V_z_obs_, and V_ss_obs_, did not differ between LPS-treated and healthy rats, nor between sexes (Fig. 7, Table S6).

In blood, oxycodone exists in both unbound (free) form and bound to plasma proteins and blood cell constituents. The fraction of unbound oxycodone in blood was 0.39 ± 0.14 (n = 7) in LPS-treated rats, which was sex independent and similar to that in healthy rats [2]. The binding and uptake of oxycodone by red blood cells was characterized by the C_b_/C_p_ ratio (Eq. 2). The mean C_b_/C_p_ of 1.15 ± 0.17 (N = 6, n = 13) in LPS-treated rats was similar to that in healthy rats (p = 0.41). Despite systemic inflammation potentially affecting hematocrit levels, no changes in Hct were observed during the LPS challenge.

Overall, LPS dramatically impacted the systemic clearance of oxycodone in female rats, being reflected in higher unbound oxycodone exposure in plasma compared to males. Yet, the fraction of unbound oxycodone in plasma and the C_b_/C_p_ ratio were sex independent and were not affected by LPS-induced inflammation.

### Alterations in intra-brain distribution of oxycodone after LPS challenge

To investigate the intra-brain distribution of oxycodone following the LPS challenge, we assessed the unbound volume of distribution, V_u,brain_ (Eq. 10). The mean V_u,brain_ obtained from oxycodone concentrations in the right striatum was 3.50 ± 0.91 mL/g brain (n = 7) in LPS-treated rats. This value was approximately two-fold higher than in healthy rats (p = 0.04) [2]. There were no differences in total oxycodone concentration between the investigated brain regions in LPS-treated rats (Fig. 4D) with a mean total oxycodone concentration in the right striatum at steady-state of 878 ± 291 ng/g brain. The total oxycodone concentration in right striatum was lower in healthy rats with a mean value of 526 ± 186 ng/g brain (p = 0.11). Thus, changes in V_u,brain_ following the LPS challenge appear to be driven by an increase in either cellular uptake and/or binding to brain parenchymal cells.

## Discussion

The current study aimed to explore the impact of LPS-induced inflammation on the systemic and CNS PK of oxycodone. Our findings highlight significant alterations in inflammatory biomarker profiles, BBB integrity, and drug transport dynamics, providing a comprehensive understanding of how endotoxemia influences oxycodone PK as a model drug for actively influxed drugs. We documented a moderate decrease in the extent of oxycodone BBB transport in an LPS-induced inflammation model, despite a dramatic increase in the paracellular transport across the BBB. Interestingly, the active net uptake of oxycodone across both the BBB and the BCSFB was preserved. The results revealed a lack of difference between K_p,uu_ values measured in the striatum, lateral ventricle, and *cisterna magna* in LPS-induced inflammation. The extent of oxycodone delivery to the striatum and *cisterna magna* was very similar, with a relative extent of 0.9–2.1, with 75% of individuals falling between 0.9–1. These results indicate that CSF is a better proxy for brain ISF in LPS-induced inflammation than in healthy rats [2].

**Fig. 8:**
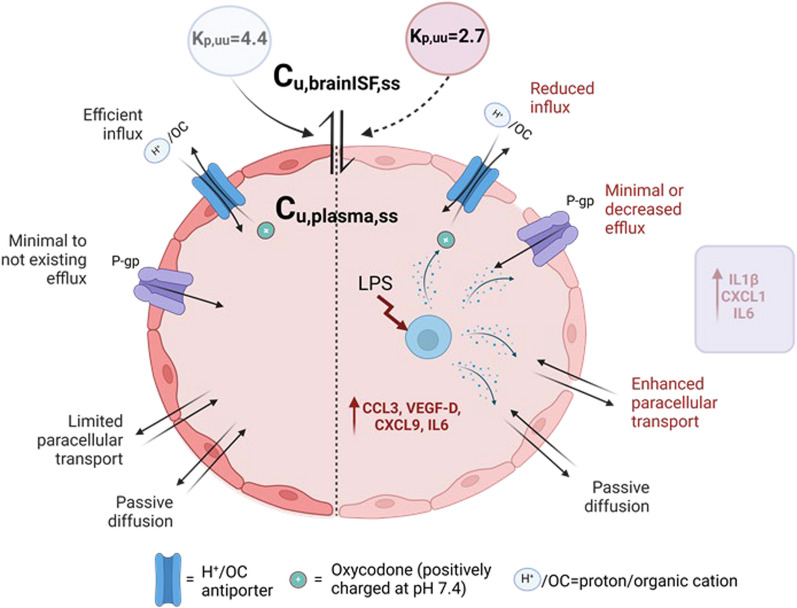
Illustration of proposed transport mechanisms of oxycodone across the blood–brain barrier (BBB) in healthy (left hand side) and LPS-treated (right hand side) rats. Oxycodone transport across the BBB occurs via passive diffusion, active transport and paracellular transport, collectively reflected in the net flux across the BBB. The unbound partition coefficient, K_p,uu,brain_, calculated as a ratio of the unbound steady-state concentration in brain ISF (C_u,brainISF,ss_) and plasma (C_u,plasma,ss_), characterizes the extent of the net transport showing dominating influx of oxycodone. LPS-induced inflammation may impact various routes of transport. Cytokines with the largest elevation in plasma and brain are indicated in the figure. Detailed explanations are provided in the Discussion section

The complexity of transport pathways across brain barriers, especially their alterations under pathological conditions, complicates the interpretation of the mechanisms driving the observed results (Fig. 8). Our evaluation of the BBB integrity, using the paracellular transport marker 4 kDa TRITC dextran, demonstrated a compromised barrier in LPS-treated rats, corroborating previous research findings [34–37]. For example, the BBB permeability measured by ^14^C-sucrose after a single 3 mg/kg LPS dose of in mice was documented to be unchanged after 4 h but increased after 24 h [35]. The significant increase in paracellular transport across the BBB is a critical observation, indicating that systemic inflammation can compromise the barrier's protective properties, potentially allowing greater penetration of both therapeutic agents and harmful low molecular weight substances. However, the paracellular transport, estimated to be 0.29% for the 4 kDa-dextran during endotoxemia (Fig. 4A-B), was overshadowed by other mechanisms that preserved the active transport of oxycodone, albeit with reduced efficiency at the BBB. The interplay between influx and efflux transporters, combined with the impact of LPS on their function, influences the extent of flux. Oxycodone is known to interact with a few drug transport systems at the BBB, with the proton-coupled organic cation (H^+^/OC) antiporter being the main contributor to the active influx [3, 4, 38]. Regarding efflux transporters, particularly P-glycoprotein (P-gp), evidence from in vitro and in vivo studies is conflicting. Some studies suggest that oxycodone might be a substrate of P-gp, as indicated by an increased brain-to-plasma concentration ratio in the mdr1 knock-out mouse model [39, 40]. However, an in vivo study in healthy rats using the P-gp inhibitor PSC833 (valspodar) showed no changes in systemic and brain PK, or its analgesic effect [41]. Additionally, Hassan et al. suggested that oxycodone might be a moderate P-gp inducer based on their in vitro and in vivo studies [39]. Moreover, the status of P-gp at the BBB in LPS-induced inflammation has been explored, revealing a biphasic impact with initial impairment up to 36 h after LPS administration, followed by induction [42–44]. We hypothesize that our model may belong to the first phase, associated with a possible reduction in P-gp function via suggested post-translational mechanisms. Analysis of existing evidence, suggests that the possible impact of P-gp on oxycodone transport in our model will likely be minimal or shifted toward reduced efflux, something that needs to be confirmed or negated in dedicated studies. This would be expected to increase the net flux of oxycodone. However, as our data show a significant moderate decrease in net influx, this may be related to decreased activity of the H^+^/OC antiporter system, combined with increased paracellular transport, following the LPS challenge.

Studies on the impact of pathological conditions on neuro-PK of the H^+^/OC antiporter system substrates are sparse. A microdialysis study using adjuvant-induced arthritis in rats reported decreased H^+^/OC antiporter activity during inflammation, with lower K_p,uu,STR_ values for diphenhydramine and memantine compared to healthy conditions [45]. Specifically, diphenhydramine uptake in the striatum was about 60% of that in healthy rats, which is similar to our findings with oxycodone. Conversely, another study on diphenhydramine in LPS-treated rats found a 2.2-fold increase in K_p,uu,STR_ without changes in BBB integrity assessed by Evans blue in a study by Kawase et al. [46]. The authors suggested an increased activity of the H^+^/OC antiporter in the LPS-induced inflammation state, something that was not observed in our study. To be noted is that LPS was administered as a single dose of 5 mg/kg i.p., and the antiporter substrate was given 6 h later, while in our study LPS was administered i.p. as three consecutive doses of 3 mg/kg per dose and oxycodone was administered 28 h after the first LPS dose. Variations in LPS dosing regimens and timing of substrate administration between the studies likely contributed to differing results. The choice of the model substrate of the antiporter may also contribute to differing conclusions about the LPS-induced impact on this system. Notably, an investigation of oxycodone disposition in the CNS using microdialysis in healthy and endotoxemic pigs revealed active uptake at the BBB, as indicated by a mean K_p,uu_ of 2.5, which showed a slight, non-significant tendency to decrease under endotoxemic conditions [14]. These findings suggest potential interspecies differences in oxycodone transport across the BBB, with more pronounced uptake observed in rats, potentially due to differences in the expression of proteins involved in the H^+^/OC antiporter system. Additionally, the BBB's dynamic response to inflammation appeared to be stronger in rats, suggesting that higher species, such as pigs, may exhibit a more stable BBB function, at least during acute endotoxemia.

Oxycodone at concentrations of 50–100 ng/mL has been documented to inhibit LPS-induced inflammatory responses, i.e., reduce cytokine production and mRNA levels, in a microglial cell culture [47]. Hence, the oxycodone administration may in itself have influenced the immune response in our study. This possible variability highlights the importance of considering individual differences in inflammation when designing therapeutic strategies involving H^+^/OC antiporter substrates. Future studies should aim to systematically explore the impact of various inflammatory states on the brain's transport mechanisms to better understand and predict drug behavior under different pathological conditions. This would help in optimizing therapeutic outcomes and minimizing adverse effects of drugs that rely on this critical transport pathway. In addition, this will help to validate the multi-substrate putative proton-coupled organic cation (H^+^/OC) antiporter system as a promising target for brain drug delivery.

In addition to documented changes in brain disposition, our study provides novel evidence of a sex-specific difference in systemic PK of oxycodone between LPS-treated and healthy rats. On average, the LPS-treated females had a clearance that was approximately half of the clearance in LPS-treated males and healthy females. Different immune response profiles in female and male rats may explain these sex-dependent changes. The LPS challenge may impact oxycodone metabolism by affecting the expression level and/or function of drug metabolizing cytochrome P450 (CYP)-enzymes, i.e., mainly CYP3A4 and CYP2D6 [48]. In a human 3D liver spheroid model treated with pathophysiologically relevant concentrations of IL-1β, IL-6 or tumor necrosis factor-α (TNF-α), pronounced decreases in the mRNA expressions of CYP3A4, and less pronounced reductions of mRNA expressions of CYP2D6 were observed [49]. In spite of sex-specific changes in systemic PK, and no sex-differences in the extent of transport across the brain barriers, there was a non-significant trend towards higher striatal exposure, governed by higher plasma exposure, in the LPS-treated female rats (Fig. S2). Studies on sex- and estrous cycle influence on oxycodone PK and its analgesic effect after oral administration of oxycodone previously reported higher systemic exposure in females [50], and higher total brain oxycodone levels and analgesia in females in the diestrous phase of the cycle [51]. However, our earlier study in healthy rats showed no sex differences in the extent of oxycodone BBB and BCSFB transport [2]. Performance of additional standardized studies focusing on sex-differences in all PK parameters, including bioavailability, in healthy and pathological conditions, will improve the reliability of the findings.

## Conclusions

This study highlights the complexity of oxycodone CNS delivery and sex-specific systemic PK changes induced by LPS administration. The LPS challenge moderately decreased oxycodone's active uptake across the BBB to the brain in a sex-independent manner, without abolishing the active uptake. This partial reduction in net uptake may still allow for similar pharmacological target engagement due to increased systemic exposure and, consequently, a comparable therapeutic response. The similar extent of BBB and BCSFB transport supports the potential use of CSF as a surrogate for brain ISF under endotoxemia conditions. These findings emphasize the importance of studying drug PK including CNS disposition in both females and males under healthy and disease conditions. Understanding these dynamics is crucial for optimizing drug therapy across different patient populations.

## Supplementary Information

Additional file 1

Additional file 2

## Data Availability

No datasets were generated or analysed during the current study.

## Abbreviations

A_brain_: Amount of drug in the brain
AUC: Area under the concentration–time curve
AUC_inf_obs_: Observed area under the concentration–time curve extrapolated to infinity
AUC_inf_D_obs_: Observed area under the concentration–time curve extrapolated to infinity and normalized by total oxycodone dose
AUC_last_: Area under the concentration–time curve from time 0 to a specified time
BBB: Blood–brain barrier
BC: Blood cells
BCSFB: Blood-cerebrospinal fluid barrier
C: Total concentration
C_b_/C_p_: Total blood-to-plasma concentration ratio
CCL: C–C motif chemokine ligand
C_er_: Total concentration in erythrocytes
CL__obs_: Observed clearance
CM: *cisterna magna*
CNS: Central nervous system
CNTN1: Contactin 1
CSF: Cerebrospinal fluid
C_ss_: Mean total concentration at steady-state
C_u_: Unbound concentration
C_u,ss_: Mean unbound concentration at steady-state
CXCL: C-X-C motif chemokine ligand
CYP: Cytochrome P
f_u,blood_: Fraction of unbound drug in the blood
f_u,plasma_: Fraction of unbound drug in plasma
H^+^/OC: Proton-coupled organic cation
IL: Interleukin
ip: Intraperitoneally
IS: Internal standard
ISF: Interstitial fluid
iv: Intravenous
K_p_: Total partition coefficient
K_p,uu_: Unbound partition coefficient
LPS: Lipopolysaccharide
LV: Lateral ventricle
NPX: Normalized protein expression
PCA: Principal component analysis
PE: Polyethylene
PEA: Proximity Extension Assay
PK: Pharmacokinetics
P-gp: P-glycoprotein
QC: Quality control
R^2^: Coefficient of determination
SD: Standard deviation
STR: Striatum
t_1/2_: Terminal half-life
TNF: Tumor necrosis factor
TRITC: Tetramethyl-rhodamine B isothiocyanate
UPLC-MS/MS: Ultraperformance liquid chromatography-tandem mass spectrometry
V_eff_: Effective plasma space of the drug
VEGF-D: Vascular endothelial growth factor D
V_er_: Erythrocyte volume
V_protein_: Apparent vascular space of plasma proteins
V_u,brain_: Apparent unbound volume of distribution in the brain
V_water_: Apparent plasma water space
V_z_obs_: Volume of distribution
V_ss_obs_: Volume of distribution at steady state
WB: Whole brain
