# Supplementary material for: Reduced oxycodone brain delivery in rats due to lipopolysaccharide-induced inflammation: microdialysis insights into brain disposition and sex-specific pharmacokinetics
Source: Fluids Barriers CNS. 2024 Dec 2;21:95. doi: 10.1186/s12987-024-00598-6 (PMC11613587; doi:10.1186/s12987-024-00598-6)
Supplement: Supplementary file 1 — Additional file 1 [file 12987_2024_598_MOESM1_ESM.pdf]

# Reduced Oxycodone Brain Delivery in Rats Due to Lipopolysaccharide-Induced Inflammation: Microdialysis Insights into Brain Disposition and Sex-Specific Pharmacokinetics

**Frida Ällgren, Margareta Hammarlund-Udenaes, Irena Loryan***

Translational Pharmacokinetics/Pharmacodynamics group (tPKPD), Department of Pharmacy, Uppsala University, Uppsala, Sweden

*Corresponding author

Irena Loryan

Email: irena.loryan@farmaci.uu.se

Translational PKPD Group, Department of Pharmacy,

Uppsala University, Box 580, 75123, Uppsala, Sweden

## 1. Supplementary Materials

### Extended Materials and Methods Section

### Chemicals

Oxycodone hydrochloride (Eur. Qual D, apl, Kungens Kurva, Sweden) was obtained from Distansapoteket, Falun, Sweden. Oxycodone hydrochloride (OxyNorm®, Mundipharma), isoflurane (Isoflo® vet., 100 %, solution, Zoetis, Finland), heparin (5000 IU/mL, Heparin LEO), buprenorphine (Temgesic®, 0.3 mg/mL solution for injection, Indivior Eur. Ltd), lidocaine (Xylocain®, 100 mg/mL, cutaneous spray, Aspen Nordic, Denmark) and sterile saline (B. Braun Medical AB, Danderyd, Sweden) were purchased from Distansapoteket Stockholm, Apoteket

AB, Stockholm, Sweden. Heparinized saline solution (100 IU heparin/mL) was prepared in-house. Sigma-Aldrich (Stockholm, Sweden) supplied oxycodone-D3 and oxycodone-D6 (Cerriliant), sodium chloride, potassium chloride, magnesium chloride, calcium dichloride, ascorbic acid, potassium dihydrogen phosphate, LPS *Salmonella enterica* serotype *typhimurium* (catalog # L6511, batch # 0000106328), RIPA Lysis Buffer (10X for Immunoprecipitation & Western Blotting; Upstate®, Millipore, Merck Life Science AB, Merck KGaA, Darmstadt, Germany) and cOmplete™Mini Protease Inhibitor Cocktail (Roche, Basel, Switzerland). Anhydrous dipotassium hydrogen phosphate, formic acid (98-100 %) and acetonitrile (gradient grade for liquid chromatography) were purchased from Merck (Darmstadt, Germany). Dental cement (Dentalon Plus; Heraeus Kulzer GmbH) was obtained from AgnTho's AB, Lidingö, Sweden. The Milli-Q Academic system (Millipore, Bedford, MA, USA; Resistance 18.2 Ohm; Millipak®Express 20 Filter, 0.22 µm) was supplied by Merck Millipore (Burlington, MA, USA). A 4 kDa tetramethyl-rhodamine B isothiocyanate (TRITC) dextran was purchased from TdB Labs AB (Uppsala, Sweden).

## Animals

Experiments were performed on drug-naïve male and female Sprague-Dawley rats (n=26) (Taconic, Lille Skensved, Denmark) of 270-330 g. The rats were group housed under a 12-hour light-dark cycle at a temperature of 20-21°C and humidity of 45-65 % with food and water *ad libitum* for one week before surgery. Guidelines from the Swedish National Board for Laboratory Animals were applied and the Animal Ethics Committee of Uppsala, Sweden (Ethical Approval Dnr. 5.8.18-12230/2019) approved the study. Pain and distress levels were evaluated in animals according to Uppsala University guidelines. Given the desired probability level ($\alpha$ = 0.05), effect size (Cohen's d = 1.2-1.5), and statistical power level (0.8), the sample

size per group required for a two-tailed t-test study was estimated to be minimally four to six (1). The study was not blinded and no randomization was applied.

The impact of the LPS challenge on various PK parameters was compared to the same parameters measured in healthy male and female Sprague-Dawley rats and reported elsewhere by Bällgren et al. (2). Studies in healthy and LPS-challenged animals were performed during the same period.

## Placement of probes and catheters

An overview of the experimental procedure is presented in Figure 1A. The first LPS dose was administered prior to the surgical implantation of probes and catheters. The surgery was performed as previously described (2). Briefly, catheters were primarily placed in the left femoral or occasionally (less than 10 % of the cases) left jugular vein (fused tubing of polyethylene (PE)-50, PE-10 and silicone), and in the left femoral artery (PE-50 and PE-10) for drug administration and blood sampling, respectively. Catheters were filled with heparinized saline (100 IU/mL) to avoid clotting and passed subcutaneously to the neck. The microdialysis probes were implanted as follows. A 10 mm CMA 20 Elite probe (CMA Microdialysis AB, Kista, Sweden) was placed in the right jugular vein using a guide cannula, and fixed to the pectoralis muscle with sutures. The CNS probes were placed in two of the three locations; striatum, the lateral ventricle and/or *cisterna magna* using a stereotaxic instrument for rats (David Kopf Instruments, Tujunga, CA). The brain stereotaxic coordinates were: striatum +0.8 mm anteroposterior and -2.7 mm lateral to bregma, and -3.8 mm dorsoventral to the surface of the brain, the lateral ventricle probe -0.9 mm anteroposterior, +1.6 mm lateral and -2.9 mm dorsoventral relative to bregma, and the *cisterna magna* -1.93 mm anteroposterior, +3.15 mm lateral and -8.1 mm dorsoventral, at an angle of 25° anterior from the dorsoventral axis,

18° lateral from the anteroposterior axis, relative to lambda (2, 3). The following CNS probes were used: a 3 mm CMA 12 Elite probe (CMA Microdialysis AB, Kista, Sweden) for striatum, and 1 mm CMA 12 Elite probes (CMA Microdialysis AB, Kista, Sweden) for the lateral ventricle and *cisterna magna*. To maintain hydration and provide pain relief 5 mL/kg of isotonic saline solution supplemented with buprenorphine of 0.01 mg/kg was administered postoperatively. Pending the experiment, the rat was housed in a CMA/120 system for freely moving animals (CMA Microdialysis AB, Kista, Sweden) with food and water *ad libitum* for approximately 27 hours to allow for recovery. Surgeries were performed between 7-10 a.m. to enable the start of the experiment at similar time points as our previous study in healthy rats (2). Surgical instruments were sterilized using a glass bead dry sterilizer (Simon Keller AG, Switzerland) at 230-250°C for 15 seconds and surgeries were performed at aseptic conditions.

## Lipopolysaccharide administration

The LPS challenge was selected as an inflammation model to investigate oxycodone PK, including brain delivery, in a disease condition. Ten rats of both sexes were administered LPS intraperitoneally (ip) at doses of 3 mg/kg at time 0, 6, and 24 h in relation to the start of surgery, according to previously published protocols in mice (4-6). Thus, the total dose of LPS administered was 9 mg/kg. LPS was dissolved in sterile saline at a concentration of 2 mg/mL and the ip injection volume of each dose was around 500 µL, depending on the rat's body weight. The LPS solution was stored in polypropylene SafeSeal micro tubes (Sarstedt AG & Co. KG, Nümbrecht, Germany) at -80°C according to the manufacturer’s recommendations. After thawing the LPS solution, it was mixed using a magnetic stirrer for 15 minutes, and at the time of the experiment, the LPS solution was vortexed to avoid injecting a sedimented solution. The constant-rate infusion of oxycodone (Dosing regimen I) was initiated at 28 h after the first LPS injection.

## Microdialysis experiment

The experimental design, including animal handling, oxycodone dosing, microdialysis experiment, and bioanalysis was reported in Bällgren et al. (2). In short, all ten rats included in the microdialysis study received a 60-minute constant-rate infusion of 0.3 mg/kg/h oxycodone (Dosing regimen I), followed by a washout period (post-infusion period) and a steady-state condition induced by a loading dose of 0.24 mg/kg given as a fast-rate infusion and a maintenance dose 0.54 mg/kg/h given as a slow-rate infusion (Dosing regimen II). Dosing regimen I enables analysis of both the rate and extent of oxycodone transport across the CNS barriers and Dosing regimen II provides a steady-state condition required for an accurate estimation of CNS drug delivery as well as plasma protein binding and brain tissue binding/uptake. Briefly, the experiment consisted of i) a 60-minute stabilization period, ii) a 60-minute constant-rate infusion, iii) a 180-minute post-infusion period, and iv) a 60-minute steady-state period (Fig. 1A). The stabilization period included probe perfusion with Ringer's solution containing 44 ng/mL of the calibrator oxycodone-D3, for assessment of probe recovery (7). The microdialysis probes were perfused with a filtered Ringer's solution, pH 7.4, at a flowrate of 1 µL/min using a CMA 400 Syringe Pump (CMA Microdialysis AB, Kista, Sweden). The oxycodone infusions were performed with a Harvard 22 pump (Harvard Apparatus Inc., Holliston, MA). Dialysate samples were collected in pre-weighed vials (polypropylene with polyurethane caps; AgnTho's, Lidingö, Sweden) in 10-minute intervals, that were thereafter weighed, capped, and stored at 6°C. Blood was sampled in heparinized (5 µL 5000 IU/mL heparin) Eppendorf tubes (polypropylene; Eppendorf, Hamburg, Germany), before the first LPS dose and at 5, 15, 25, 45, 55, 75, 85, 115, 175 and 275 minutes after initiation of Dosing regimen I. A maximum of 180 µL per blood sample and minimum 10 mL of terminal blood were collected from each rat. The blood samples were centrifuged

(MicroStar12 centrifuge; VWR International AB, Stockholm, Sweden; or IEC Centra CL2, Labora; IEC international equipment company, Needham Heights, MA, USA) and plasma was transferred to new Eppendorf tubes and stored at -20°C. One hemisphere and striatum from both hemispheres were collected after terminal blood sampling; the right striatum as a reference for probe placement, and the left as a reference without probe. The brains were visually examined for confirmation of correct probe placements and notations of possible macro changes related to the implantation of the probes. The brain samples were stored covered by aluminum foil in polystyrene cups (VWR International, Radnor, USA) at -80°C.

## *In vivo* recovery calculation

To correct for the incomplete recovery across the probe membranes and enable estimation of unbound oxycodone concentrations at probe sites, retrodialysis by calibrator was performed *in vivo* throughout the experiment (7). The method assumes that the probe loss of the calibrator, here oxycodone-D3, is equal to the recovery of the drug, which is a valid assumption as oxycodone and oxycodone-D3 are almost identical molecules. The individual probe recovery was calculated as follows:

$$Recovery = {(C_{in} - C_{out})}/{C_{in}} \quad (1)$$

where $C_{in}$ is the mean calibrator concentration in the perfusion solution entering the probe, sampled from the perfusion syringes before and after the experiment. $C_{out}$ is the mean concentration of the calibrator in the dialysate samples leaving the probe, collected from each probe throughout the experiment. Mean recoveries based on individual recoveries were calculated for the blood probe (10 mm), striatum probe (3 mm) and the lateral ventricle and *cisterna magna* probe (1 mm) as the recovery is dependent on the probe length. As the recovery of probes with shorter membranes is more sensitive to fluctuations in recovery,

mean recoveries were used for the estimations in striatum (3 mm), and both lateral ventricle and *cisterna magna* (1 mm). To determine which values to include in the mean estimations and exclude unrealistic recovery values, previously published oxycodone recoveries were used as references (8, 9). Recoveries within two SD around the means of the previously published values were included. For the blood probes with longer membranes, the values were higher and, therefore, not as sensitive to fluctuations. Hence, individual (i.e., per rat) recoveries were used for estimations of unbound blood concentrations.

## Measurement of oxycodone partition into blood cells after LPS challenge

The blood-to-plasma concentration ratio ($C_b/C_p$) of oxycodone has been reported to be above unity (2, 8). The status of oxycodone red blood cell partitioning as well as hematocrit were investigated in the LPS-treated rats (n=6) (2). The $C_b/C_p$ ratio was calculated as follows, according to Tozer (10):

$$C_b/C_p = 1 - Hct + Hct \times (C_{BC}/C_{plasma}) \qquad (2)$$

where $C_{BC}$, and $C_{plasma}$ are total concentrations in blood cells (BC), and plasma, respectively, and Hct is the hematocrit.

## Bioanalysis

The bioanalysis of dialysate, plasma, blood cells, and brain samples as well as the respective standards, quality controls (QCs), and blanks, was performed according to the previously described protocol (2). In brief, the quantification of oxycodone, the calibrator oxycodone-D3 and the internal standard (IS) oxycodone-D6 was performed by ultraperformance liquid chromatography-tandem mass spectrometry (UPLC-MS/MS) using an Acquity Ultra-Performance Liquid Chromatography system (Waters Corporation, Milford, Massachusetts,

USA), coupled to a Xevo TQ-S Micro mass spectrometer (Waters Corporation, Milford, Massachusetts, USA). The column and pre-column were AQUITY UPLC BEH C18-column, 1.7 µm, 2.1 x 50 mm (Waters Corporation, Milford, Massachusetts, USA) and AQUITY UPLC BEH C18 VanGuard Pre-Column (Waters Corporation, Milford, Massachusetts, USA). Gradient elution was applied, with mobile phases A and B consisting of 0.1 % formic acid in MilliQ water and 0.1 % formic acid in acetonitrile, respectively. The transition modes of oxycodone, oxycodone-D3 and oxycodone-D6 were 316.11 → 298.1 *m/z*, 319.11 → 301.1 *m/z*, and 322.18 → 304.1 *m/z*, respectively. MassLynx version 4.2, and TargetLynx (Waters Corporation, Milford, Massachusetts, USA) were used for the quantification of analytes. The calibration curves in the different matrices included standards of 0.5-150 ng/mL, and QCs of 2, 25, and 75 ng/mL. Linear regression and a weighing function of $1/X^2$ were applied to obtain calibration curves.

## Proteomic analysis

To confirm the achievement of inflammation induced by LPS, proteomic analysis was performed on randomly selected animals using the Target 96 Mouse Exploratory panel (Olink Proteomics AB, Uppsala, Sweden), which includes quantification of well-known inflammation biomarkers (Table S1). The analysis is based on Proximity Extension Assay (PEA) technology, where oligonucleotide labeled antibody probe pairs are binding to their respective target protein present in the sample. According to the manufacturer, the Target 96 Mouse Exploratory panel can be extended to analyze rat proteins.

The proteins were quantified in plasma and brain, i.e., right striatum (brain probe placement) and left striatum (contralateral side with no probe placement). An overview of the four study groups (n=4 per group) and collection of plasma and brain samples used for the proteomic

analysis is presented in Figure 2A. Briefly, samples from the following groups were included: A. LPS-treated rats in the microdialysis oxycodone study, B. healthy rats in the microdialysis oxycodone study, C. LPS-treated controls without microdialysis, and D. healthy controls without microdialysis or LPS challenge. All groups included an even distribution of females and males. Rats in the A and B groups had undergone all steps of the microdialysis study (Fig. 1A) with or without the LPS challenge, respectively. Rats in the C and D groups only went through acclimatization, with or without LPS challenge, respectively. In the C group, terminal samples were collected approximately eight hours after the third LPS dose, to match the terminal sampling time point of the rats included in the microdialysis study (A).

Plasma samples from LPS-treated rats were collected right after the first LPS dose, before starting implantation of microdialysis probes (Group A: A baseline), and after administration of the third LPS dose (Group A), as well as after administration of the third LPS dose to rats not undergoing microdialysis (C: LPS_3 doses_no surgery). Plasma samples from healthy rats were collected at the steady-state phase of microdialysis (B: Healthy control_surgery) and in healthy controls (D: Healthy control_no surgery).

Brain samples from LPS-treated rats were collected terminally at the end of the steady-state phase of oxycodone infusion (A: LPS_surgery_no probe, LPS_surgery_with probe), and after administration of the third LPS dose (C: LPS control_no surgery). Brain samples from healthy rats were collected at the steady-state phase of microdialysis (B: Healthy_surgery_no probe and Healthy_surgery_with probe) and in control rats (D: Healthy control_no surgery).

The plasma and brain samples from striatal regions were prepared according to Olink's instructions. Plasma samples were transferred to a 96-well plate (96 fast PCR full-skirt, Sarstedt AG & Co. KG, Nümbrecht, Germany), sealed by MicroAmp$^{TM}$ Optical adhesive film

(PCR/real-time PCR compatible) and placed in -80°C pending analysis. Brain tissue samples were lysed using the lysis buffer working solution supplemented with protease inhibitors 1:20 (w:v) and homogenized on ice using an ultrasonicator (Sonics vibra cell, Chemical instruments AB; Sonic materials Inc., Newtown, USA). Thereafter, the brain homogenate samples were centrifuged at 10,000 rpm for 5 minutes in cold conditions, and the supernatant was transferred to low protein-binding tubes (Sarstedt AG & Co. KG, Nümbrecht, Germany). The total protein content was measured in each sample using a Spectrophotometer (DeNovix DS-11, Wilmington, DE, USA), and diluted to 0.5 mg protein/mL using lysis buffer supplemented with protease inhibitors, if needed. Brain homogenate samples were then transferred to a 96-well plate, sealed by MicroAmp™ Optical adhesive film and placed in -80°C pending analysis. Both plasma samples and brain homogenate samples were randomly organized on their respective 96-well plate, and pseudonymized sample identification numbers were used for each sample. Samples were analyzed at Olink Proteomics AB lab in Uppsala, Sweden. Data were reported using a Normalized Protein Expression (NPX) unit. NPX is on a log2 scale, i.e., a larger number represents a higher protein level in the sample, typically with the background level at around zero.

### Assessment of the BBB integrity

The integrity of the BBB was assessed in LPS-treated (n=4) and healthy (n=3) rats by evaluating the extent of a 4 kDa TRIC dextran transport across the barrier as previously described (11, 12). Precautions were taken throughout the experiment and bioanalysis to prevent potential degradation of dextran due to light sensitivity. Rats underwent surgery one day before the experiment. The surgery included femoral vein and artery catheterization for dextran administration and blood sampling, respectively. In addition, the placement of a microdialysis probe guide into the right striatum of all animals was

performed, aiming to mimic the experimental conditions during the microdialysis studies. LPS was administered as described above. On the day of the experiment, an iv infusion of dextran was administered at a dose of 400 mg/kg over 5 minutes using a Harvard 22 pump (Harvard Apparatus Inc., Holliston, MA). Blood was sampled before the infusion and 5 minutes after the start of the infusion. Terminally, blood was sampled by heart puncture, followed by transcardial perfusion using 0.9 % NaCl at a rate of 10 mL/min for 2 minutes. Before the blood was centrifuged at 10,000 rpm for 5 minutes, it was allowed to coagulate for 15-20 minutes at room temperature. Thereafter, serum was collected, diluted 1:9 (v:v) in MilliQ water, and stored at 6 °C until bioanalysis. The brain was isolated and dissected into the two hemispheres, followed by microdissection of the left and right striatum (Fig. 1B). Left and right striatal areas, and one remaining hemisphere (referred to as the whole brain, WB) were weighed and homogenized 1:3 (w:v) in MilliQ water using a 4-Place Beads Homogenizer (VWR, Stockholm, Sweden). Samples were centrifuged at 14800 rpm for 1 h at 4 °C, followed by supernatant collection.

For quantification of 4 kDa TRITC dextran in samples, the standards in diluted serum (1:9, v:v, with MilliQ water) and the supernatant obtained from blank brain homogenate (1:3, w:v, in MilliQ water) ranged from 5-1250 µg/mL and 0.05 – 365 µg/g brain tissue, respectively, were used. Quantitative analysis of dextran in the samples was conducted within four hours following the end of the experiment. Samples, standards, and blanks in the respective matrices, were loaded onto a 96-well microplate (solid black polystyrene, half area, flat bottom, Corning® Incorporated, Corning, NY, USA). Each sample was analyzed in duplicates. Tecan Spark® Multimode Microplate Reader (Tecan Group Ltd., Männedorf, Switzerland) was used to measure the intensity of the fluorescent TRITC dextran at an excitation wavelength of 550 nm and an emission wavelength of 571 nm. The highest

standard was used to optimize the signal. Brain-to-serum concentration ratio was estimated using terminal blood and brain samples.

## Data analysis for PK parameters

For the estimation of unbound oxycodone concentrations at the sites of probe placement, the concentrations measured in the dialysate samples were divided by recovery of the probe, according to the following equation:

$$C_u = {C_{dialysate}}/{Recovery} \quad (3)$$

where $C_u$ is the unbound concentration at the probe location, $C_{dialysate}$ is the concentration in the dialysate sample, and Recovery (Eq. 1) is the calibrator recovery across the probe membrane.

For estimation of total oxycodone concentrations in blood ($C_{blood}$) from the measured total concentrations in plasma ($C_{plasma}$), the total concentration in plasma was multiplied with $C_b/C_p$, according to the following equation:

$$C_{blood} = C_{plasma} \times {C_b}/{C_p} \quad (4)$$

PK parameters, including the area under the concentration-time curve extrapolated to infinity ($AUC_{inf\_obs}$) and from time 0-180 minutes ($AUC_{last}$), clearance ($CL_{\_obs}$), volume of distributions ($V_{z\_obs}$ and $V_{ss\_obs}$) and terminal half-life ($t_{1/2}$) were estimated using Phoenix 64 WinNonLin (Certara, New Jersey, USA) as previously described (2). The unbound concentrations at steady-state ($C_{u,ss}$) were calculated as the mean concentration in samples collected at 35, 45, and 55 minutes after initiation of Dosing regimen II. The total blood concentrations at steady-state were calculated from at least two samples collected a minimum of 35 minutes after initiation of Dosing regimen II.

The fraction of unbound drug in the blood ($f_{u,blood}$) was calculated as follows:

$$f_{u,blood} = {C_{u,blood,ss}}/{C_{tot,blood,ss}} \quad (5)$$

where $C_{u,blood,ss}$ is the mean unbound concentration in blood at steady-state and $C_{tot,blood,ss}$ is the mean total concentration in blood at steady-state.

The unbound partition coefficients, describing the extent of drug delivery to striatum ($K_{p,uu,STR}$), lateral ventricle ($K_{p,uu,LV}$) and *cisterna magna* ($K_{p,uu,CM}$) were estimated as follows:

$$K_{p,uu} = {AUC_{u,brain}}/{AUC_{u,blood}} \quad (6)$$

and

$$K_{p,uu} = {C_{u,brain,ss}}/{C_{u,blood,ss}} \quad (7)$$

where $AUC_{u,brain}$ and $C_{u,brain,ss}$ are AUC and the mean steady-state unbound concentration in brain ISF in striatum or CSF in the lateral ventricle or *cisterna magna*, respectively; $AUC_{u,blood}$ and $C_{u,blood,ss}$ are AUC and the mean steady-state unbound concentration in blood.

To compare the extent of drug delivery between two CNS sites, the relative extent was determined based on the ratio of the respective $K_{p,uu}$ values:

$$Relative\ extent_{CNS1/CNS2} = {K_{p,uu,CNS1}}/{K_{p,uu,CNS2}} \quad (8)$$

The partition coefficient between total brain and blood concentrations, $K_{p,brain}$, was estimated as follows:

$$K_{p,brain} = {C_{tot,brain,ss}}/{C_{tot,blood,ss}} \quad (9)$$

where $C_{tot,brain,ss}$ is the total concentration in the brain (right or left striatum, or whole brain) at steady-state, and $C_{tot,blood,ss}$ is the total concentration in blood at steady-state.

The apparent unbound volume of distribution in the brain ($V_{u,brain}$), describing the intra-brain distribution, was calculated as follows:

$$V_{u,brain} = A_{brain} / C_{u,brainISF} \quad (10)$$

where $A_{brain}$ is the amount of drug in the right striatum corrected for the residual blood in the brain (Eq. 11), and $C_{u,brainISF}$ is the unbound concentration in brain ISF.

$$A_{brain} = \frac{C_{brain} - (V_{eff} \times C_{plasma}) - (V_{er} \times C_{er})}{1 - V_{water} - V_{er}} \quad (11)$$

where $C_{brain}$, $C_{plasma}$ and $C_{er}$ are the total drug concentrations in the brain, plasma, and erythrocytes, respectively. $V_{er}$ and $V_{water}$ are the erythrocyte volume and the apparent plasma water space, respectively. Values for $V_{er}$ of 2.13 µL/g brain and $V_{water}$ of 10.3 µL/g brain were used (13). $V_{eff}$ is the effective plasma space of the drug, calculated as follows:

$$V_{eff} = f_{u,plasma} \times V_{water} + (1 - f_{u,plasma}) \times V_{protein} \quad (12)$$

where $f_{u,plasma}$ is the fraction of unbound drug in plasma, $V_{water}$ is the apparent plasma water space and $V_{protein}$ is the apparent vascular space of plasma proteins. The $f_{u,plasma}$ value used was 0.87 (2), and the $V_{protein}$ value used was 7.99 µL/ g brain (13).

## Inclusion and exclusion criteria

Different inclusion and exclusion criteria for the different parts of the study were applied. The criteria for inclusion in the microdialysis study were: healthy rats of both sexes with a body weight within 270-330 g, and successful catheterization of blood vessels and placements of

probes. Throughout the study, Uppsala University assessment guidelines for pain and distress in experimental animals were used to determine the adequate health status of the rat. Two experiments were terminated earlier than intended due to the poor health status of the rats observed after administration of LPS. Acceptance and inclusion criteria for the bioanalysis were as follows: i) the variability in the precision and the accuracy within ±15 %, except at the lower limit of quantification level (0.5 ng/mL) with the accuracy of ±20 % was allowed; ii) the range for the IS recovery in standards and QCs was 80-120 %; and iii) the coefficient of determination ($R^2$) of the calibration curves was set to be above 0.99. During PK data analysis, the following inclusion criteria were applied: a maximum of 40 % of the data points were allowed to be missing when estimating AUC; and the extrapolated area (from the last time point to infinity) was not allowed to exceed 20 % of the total estimated area (from time 0 to infinity) for estimation of the $AUC_{inf\_obs}$. If the extrapolated area was larger than 20 % of the total estimated area, the AUC from time 0 to the last time point ($AUC_{last}$) was used for further calculations, e.g., $K_{p,uu}$. The two terminated experiments did not meet the criterion of a maximum of 40 % of the intended data points missing. Hence, the obtained $AUC_{last}$ was used for further calculations, where appropriate.

## Statistical analysis

GraphPad Prism version 9.0.0 for Windows (GraphPad Software, San Diego, California USA, http://www.graphpad.com) was used for statistical analyses. All data are presented as mean ± standard deviation (SD). Shapiro-Wilk normality test was used to test normal distribution, and if passed, parametric statistical tests were used for data analysis. An unpaired, two-tailed t-test was used for comparisons of PK parameters and concentrations between LPS-treated and healthy rats. One-way ANOVA and Tukey's multiple comparison tests were used to compare PK parameters by CNS location (striatum, lateral ventricle, *cisterna magna*) in LPS-

treated or healthy rats. Two-way ANOVA and Šídák's or Tukey's multiple comparison tests were used to compare PK parameters by CNS location and by method of estimation in LPS-treated and healthy rats, and between the four groups (LPS-treated and healthy females and males). GraphPad Prism automatically analyzes data sets with missing values, by fitting a mixed effects model, as ANOVA cannot be used for data sets with missing values. The correlation between $K_{p,uu}$ and time, and $K_{p,uu}$ and concentration, respectively, were assessed with Pearson correlation coefficients obtained by computing two-tailed correlations. Significant differences were indicated by $p<0.05$. Data from the proteomic analysis using the Target 96 Mouse Exploratory Panel from Olink were analyzed by means of ANOVA F-test with p-value correction performed using the Benjamini-Hochberg method followed by post-hoc analysis using Tukey's test. Principal component analysis (PCA) was performed on plasma and brain proteomic datasets using SIMCA 17 (Sartorius, Sweden).

## Tables (Supplementary material)

### *S1*

**Table S1. Biomarker proteins included in the Target 96 Mouse Exploratory analysis panel.**

| Olink® Target 96 Mouse Exploratory | | |
|---|---|---|
| **UniprotID Human** | **UniprotID Mouse** | **Protein name** |
| P30882 | P13501 | C-C motif chemokine 5 (CCL5) |
| O88393 | Q03167 | Transforming growth factor beta receptor type 3 (TGFBR3) |
| P55095 | P01275 | Glucagon (GCG) |
| Q4V9Z5 | Q6UXD5 | Seizure 6-like protein 2 (SEZ6L2) |
| Q9CWS0 | O94760 | N(G),N(G)-dimethylarginine dimethylaminohydrolase 1 (DDAH1) |
| P70236 | P52564 | Dual specificity mitogen-activated protein kinase kinase 6 (MAP2K6) |
| Q8R5A3 | Q7Z5R6 | Amyloid beta A4 precursor protein-binding family B member 1-interacting protein (APBB1IP) |

| | | |
|---|---|---|
| Q6ZQA6 | O75054 | Immunoglobulin superfamily member 3 (IGSF3) |
| Q8BTW9 | O96013 | Serine/threonine-protein kinase PAK 4 (PAK4) |
| Q99KJ8 | Q13561 | Dynactin subunit 2 (DCTN2) |
| P26323 | Q01543 | Friend leukemia integration 1 transcription factor (FLI1) |
| P97946 | O43915 | Vascular endothelial growth factor D (VEGFD) |
| P31240 | P01127 | Platelet-derived growth factor subunit B (PDGF subunit B) |
| P47931 | P19883 | Follistatin (FS) |
| Q9Z109 | Q96IQ7 | V-set and immunoglobulin domain-containing protein 2 (VSIG2) |
| Q9R000 | Q9UKP3 | Melusin (ITGB1BP2) |
| P11152 | P06858 | Lipoprotein lipase (LPL) |
| P11103 | P09874 | Poly [ADP-ribose] polymerase 1 (PARP-1) |
| Q61982 | Q9UM47 | NEUgenic locus notch homolog protein 3 (Notch 3) |
| P12960 | Q12860 | Contactin-1 (CNTN1) |
| Q09163 | P80370 | Protein delta homolog 1 (DLK-1) |
| P25446 | P25445 | Tumor necrosis factor receptor superfamily member 6 (FAS ) |
| Q99JW5 | P16422 | Epithelial cell adhesion molecule (Ep-CAM) |
| P70677 | P42574 | Caspase-3 (CASP-3) |
| O08746 | O00339 | Matrilin-2 (MATN2) |
| P47713 | P47712 | Cytosolic phospholipase A2 (PLA2G4A) |
| Q9ERB0 | O95721 | Synaptosomal-associated protein 29 (SNAP29) |
| Q7TQN3 | Q8TEU8 | WAP, Kazal, immunoglobulin, Kunitz and NTR domain-containing protein 2 (WFIKKN2) |
| Q9JLL0 | Q9NZV1 | Cysteine-rich motor NEUn 1 protein (CRIM1) |
| O89017 | Q99538 | Legumain (LGMN) |
| Q69Z26 | Q8IWV2 | Contactin-4 (CNTN4) |
| Q9EQC7 | O95633 | Follistatin-related protein 3 (FSTL3) |
| O89023 | O14773 | Tripeptidyl-peptidase 1 (TPP1) |
| P99029 | P30044 | Peroxiredoxin-5, mitochondrial (PRDX5) |
| Q9Z0T9 | P18564 | Integrin beta-6 (ITGB6) |
| Q80UG2 | Q9HCM2 | Plexin-A4 (PLXNA4) |
| P12850 | P09341 | C-X-C motif chemokine 1 (CXCL1) |
| P10855 | P10147 | C-C motif chemokine 3 (CCL3) |
| P08505 | P05231 | Interleukin-6 (IL6) |
| P20826 | P21583 | Stem cell factor (SCF) |
| P10148 | P13500 | Monocyte chemotactic protein 1 (MCP-1) |
| O08712 | O00300 | Osteoprotegerin (OPG) |

| | | |
|---|---|---|
| P01582 | P01583 | Interleukin-1 alpha (IL-1 alpha) |
| P18340 | Q07325 | C-X-C motif chemokine 9 (CXCL9 ) |
| P04202 | P01137 | Latency-associated peptide transforming growth factor beta-1 (LAP TGF-beta-1) |
| O54907 | O43508 | Tumor necrosis factor (Ligand) superfamily, member 12 (TWEAK) |
| O89093 | P78556 | C-C motif chemokine 20 (CCL20) |
| P06804 | P01375 | Tumor necrosis factor (TNF) |
| P04401 | P05113 | Interleukin-5 (IL5) |
| P18893 | P22301 | Interleukin-10 (IL10) |
| Q62386 | Q16552 | Interleukin-17A (IL-17A) |
| O35625 | O15169 | Axin-1 (AXIN1) |
| P20181 | P20783 | NEUtrophin-3 (NT-3) |
| P48540 | P39905 | Glial cell line-derived NEUtrophic factor (GDNF) |
| Q08048 | P14210 | Hepatocyte growth factor (HGF) |
| P48030 | P01135 | Transforming growth factor alpha (TGF-alpha) |
| Q8R373 | Q9H6B4 | CXADR-like membrane protein (CLMP) |
| P58058 | O95544 | NAD kinase (NADK) |
| Q9ER65 | Q9H4D0 | Calsyntenin-2 (CLSTN2) |
| Q9D6N1 | Q8N1Q1 | Carbonic anhydrase 13 (CA13) |
| Q8VCF1 | Q8WVQ1 | Soluble calcium-activated nucleotidase 1 (CANT1) |
| Q9DCL8 | P41236 | Protein phosphatase inhibitor 2 (PPP1R2) |
| P17183 | P09104 | Gamma-enolase (ENO2) |
| Q8BVI4 | P09417 | Dihydropteridine reductase (QDPR) |
| Q9EQX0 | Q9UBU3 | Appetite-regulating hormone (GHRL) |
| P30561 | P35869 | Aryl hydrocarbon receptor - mouse (Ahr) |
| Q8CD15 | Q8IUF8 | Ribosomal oxygenase 2 - mouse (Riox2) |
| Q8K4B4 | Q5VWK5 | Interleukin-23 receptor - mouse (Il23r) |
| P01587 | P04141 | Granulocyte-macrophage colony-stimulating factor - mouse (Csf2) |
| Q7TNI7 | Q96PD4 | Interleukin-17F - mouse (Il17f) |
| P10749 | P01584 | Interleukin-1 beta - mouse (Il1b) |
| Q9CR75 | Q9NP84 | Tumor necrosis factor receptor superfamily member 12A (TNFRSF12A) |
| Q6PCX7 | Q96B86 | Repulsive guidance molecule A (RGMA) |
| P97785 | P56159 | GDNF family receptor alpha-1 (GFR-alpha-1) |
| Q9R1V7 | O75077 | Disintegrin and metalloproteinase domain-containing protein 23 (ADAM 23) |
| Q8BLU0 | O43155 | Leucine-rich repeat transmembrane protein FLRT2 (FLRT2) |
| Q61288 | P37023 | Serine/threonine-protein kinase receptor R3 (SKR3) |

| | | |
|---|---|---|
| P97326 | P55285 | Cadherin-6 (CDH6) |
| Q8BX35 | Q9HAV5 | Tumor necrosis factor receptor superfamily member 27 (EDA2R) |
| Q8BYI9 | Q92752 | Tenascin-R (TN-R) |
| Q00493 | P16870 | Carboxypeptidase E (CPE) |
| P07091 | P26447 | Protein S100-A4 (S100A4) |
| Q61865 | Q16674 | Melanoma-derived growth regulatory protein (MIA) |
| P18406 | O00622 | Protein CYR61 (CYR61) |
| Q61527 | Q15303 | Receptor tyrosine-protein kinase erbB-4 (ErbB4/HER4) |
| Q61483 | O00548 | Delta-like protein 1 (DLL1) |
| O54775 | O95388 | WNT1-inducible-signaling pathway protein 1 (WISP-1) |
| Q04736 | P07947 | Tyrosine-protein kinase Yes (YES1) |
| Q9R1E0 | Q12778 | Forkhead box protein O1 (FOXO1) |
| P07321 | P01588 | Erythropoietin (EPO) |
| P48787 | P19429 | Troponin I, cardiac muscle (TNNI3) |
| Q8CGN5 | O60240 | Perilipin-1 (PLIN1) |

*S2*

**Table S2. Brain-to-serum concentration ratios of the 4 kDa TRITC dextran in left and right striatum (STR), and whole brain (WB) in LPS-treated (n=4) and healthy (n=3) rats.**

| **Location/Group** | *Healthy* | *LPS-treated* |
|---|---|---|
| **STR L** | 0.00038±0.0002 | 0.0013±0.0007 |
| **STR R** | 0.00050±0.0002 | 0.0029±0.0010 |
| **WB R** | 0.0012±0.0003 | 0.0025±0.0003 |

Mean ± SD. Statistical comparisons are presented in Table S3.

*S3*

**Table S3. Statistical test details of the 4 kDa TRITC dextran $K_{p,brain}$ in left and right striatum (STR) and whole brain (WB) in LPS-treated and healthy rats.**

| Repeated measures two-way ANOVA and Šídák's multiple comparisons test | | | | | |
|---|---|---|---|---|---|
| **ANOVA results** | | | | | |
| | **Region x Healthy-LPS** | **Region** | **Healthy-LPS** | **Subject** | **Residual** |
| **Source of Variation** | | | | | |
| **% of total variation** | 8.509 | 16.36 | 51.59 | 4.122 | |
| **P value** | 0.1278 | 0.0566 | 0.0005 | 0.7747 | |
| **P value summary** | ns | ns | *** | ns | |
| **Significant?** | No | No | Yes | No | |
| **Geisser-Greenhouse's epsilon** | | 0.6832 | | | |
| **ANOVA table** | | | | | |
| **SS** | 0.000002061 | 4E-06 | 0.00001249 | 9.983E-07 | 0.000004049 |
| **DF** | 2 | 2 | 1 | 5 | 10 |
| **MS** | 0.00000103 | 2E-06 | 0.00001249 | 1.997E-07 | 4.049E-07 |
| **F (DFn, DFd)** | F (2, 10) = 2,545 | F (1,366, 6,832) = 4,893 | F (1, 5) = 62,58 | F (5, 10) = 0,4931 | |
| **P value** | P=0,1278 | P=0,0566 | P=0,0005 | P=0,7747 | |
| **Multiple comparisons** | | | | | |
| | **Healthy - LPS** | **STR L** | **STR R** | **WB R** | |
| **Šídák's multiple comparisons test** | | | | | |
| **Mean Diff,** | | -9E-04 | -0.002421 | -0.001336 | |
| **95,00% CI of diff,** | | -0,002628 to 0,0007896 | -0,004446 to -0,0003959 | -0,002452 to -0,0002200 | |
| **Below threshold?** | | No | Yes | Yes | |
| **Summary** | | ns | * | * | |
| **Adjusted P Value** | | 0.2486 | 0.0285 | 0.0282 | |
| **Test details** | | | | | |
| **Mean 1** | | 0.000388 | 0.0005029 | 0.001164 | |
| **Mean 2** | | 0.001307 | 0.002924 | 0.0025 | |
| **Mean Diff,** | | -0.0009191 | -0.002421 | -0.001336 | |
| **SE of diff,** | | 0.0003951 | 0.0004841 | 0.0002695 | |
| **N1** | | 3 | 3 | 3 | |
| **N2** | | 4 | 4 | 4 | |
| **t** | | 2.326 | 5.001 | 4.958 | |
| **DF** | | 3.459 | 3.632 | 3.689 | |

S4

**Table S4**. **Statistical test details of comparisons of $K_{p,uu}$ in STR, LV, and CM between LPS-treated and healthy rats.**

| **Two-way ANOVA and Šídák's multiple comparison test results** | | | | |
|---|---|---|---|---|
| **Test details** | **Healthy - LPS** | **STR** | **LV** | **CM** |
| **Predicted (LS) mean 1** | | 4.44 | 3.405 | 2.68 |
| **Predicted (LS) mean 2** | | 2.719 | 2.474 | 2.236 |
| **Predicted (LS) mean diff.** | | 1.721 | 0.931 | 0.4435 |
| **SE of diff.** | | 0.3439 | 0.4456 | 0.5185 |
| **N1** | | 17 | 10 | 9 |
| **N2** | | 10 | 6 | 4 |
| **DF** | | 50 | 50 | 50 |
| **95.00% CI of diff.** | | 0.8716 to 2.571 | -0.1698 to 2.032 | -0.8375 to 1.725 |
| **Adjusted P Value** | | <0.0001 | 0.1202 | 0.7801 |

$K_{p,uu}$ in striatum (STR), lateral ventricle (LV) and *cisterna magna* (CM) were estimated using AUC in LPS-treated and healthy rats. Data are presented in Figure 6 and Table 1.

*S5*

**Table S5. Statistical test details of comparisons of $K_{p,uu,STR}$ between the groups: LPS-treated and healthy female and male rats.**

| Two-way ANOVA and Šídák's multiple comparison test results | | | | | | |
|---|---|---|---|---|---|---|
| **Test details** | **Healthy:F vs. Healthy:M** | **Healthy:F vs. LPS:F** | **Healthy:F vs. LPS:M** | **Healthy: M vs. LPS:F** | **Healthy: M vs. LPS:M** | **LPS:F vs. LPS:M** |
| **Predicted (LS) mean 1** | 4.193 | 4.193 | 4.193 | 4.613 | 4.613 | 2.357 |
| **Predicted (LS) mean 2** | 4.613 | 2.357 | 2.959 | 2.357 | 2.959 | 2.959 |
| **Predicted (LS) mean diff.** | -0.4195 | 1.836 | 1.234 | 2.255 | 1.653 | -0.6019 |
| **SE of diff.** | 0.4708 | 0.5988 | 0.5315 | 0.5652 | 0.4933 | 0.6167 |
| **N1** | 7 | 7 | 7 | 10 | 10 | 4 |
| **N2** | 10 | 4 | 6 | 4 | 6 | 6 |
| **DF** | 23 | 23 | 23 | 23 | 23 | 23 |
| **95.00% CI of diff.** | -1.774 to 0.9350 | 0.1128 to 3.558 | -0.2955 to 2.763 | 0.6290 to 3.881 | 0.2338 to 3.072 | -2.376 to 1.172 |
| **Adjusted P Value** | 0.9443 | 0.0324 | 0.1645 | 0.0035 | 0.0165 | 0.9167 |

$K_{p,uu}$ in striatum (STR) in LPS-treated and healthy female (F) and male (M) rats. Data are presented in Figure 6 and Table 1.

*S6*

**Table S6. Sex-dependent unbound blood pharmacokinetic parameter estimates of oxycodone in LPS-treated rats.**

| Parameter | Unit | Both sexes | Females | Males |
|---|---|---|---|---|
| **$C_{u,blood,ss}$** | ng/mL | 80.4±23.9 | 89.9±37.3 | 73.3±7.1 |
| **$AUC_{inf\_D\_obs}$** | $\frac{min \times \mu g \times mL^{-1}}{mg/kg}$ | 15.2±8.0 (n=8) | 22.1±8.8 (n=3) | 11.1±4.1 (n=5) |
| **$AUC_{inf\_obs}$** | $min \times \mu g \times mL^{-1}$ | 4.8±2.6 (n=6) | 6.6±2.6 (n=3) | 2.8±0.2 (n=3) |
| **$CL_{\_obs}$** | $mL \times min^{-1} \times kg^{-1}$ | 79.7±32.8 (n=8) | 49.96±18.0 (n=3) | 97.5±25.7 (n=5) |
| **$V_{ss\_obs}$** | $mL \times kg^{-1}$ | 4828±1423 (n=8) | 4728±1978 (n=3) | 4889±1256 (n=5) |
| **$V_{z\_obs}$** | $mL \times kg^{-1}$ | 4852±1496 (n=8) | 4166±1574 (n=3) | 5263±1454 (n=5) |
| **$t_{1/2}$** | *min* | 46.0±16.8 (n=8) | 60.6±21.8 (n=3) | 37.3±0.82 (n=5) |

Parameters were estimated from data obtained by Dosing regimen I. Mean±SD. $AUC_{inf\_D\_obs}$ is the AUC from time 0 extrapolated to infinity normalized by oxycodone dose in mg/kg (Table S7). $CL_{\_obs}$ is the clearance (See statistical details in Table S8). $V_{ss\_obs}$ and $V_{z\_obs}$ are the volumes of distribution at steady-state (for non-steady-state data) and based on the terminal phase, respectively. $t_{1/2}$ is the terminal half-life (See statistical details in Table S9).

*S7*

**Table S7. Statistical test details of comparisons of systemic $AUC_{inf\_D\_obs}$ in LPS-treated and healthy female and male rats.**

| Two-way ANOVA and Tukey's multiple comparison test results | | | | | | |
|---|---|---|---|---|---|---|
| **Test details** | **Healthy:F vs. LPS:F** | **Healthy:F vs. Healthy:M** | **Healthy:F vs. LPS:M** | **LPS:F vs. Healthy: M** | **LPS:F vs. LPS:M** | **Healthy: M vs. LPS:M** |
| **Predicted (LS) mean 1** | 10579 | 10579 | 10579 | 22082 | 22082 | 10070 |
| **Predicted (LS) mean 2** | 22082 | 10070 | 11111 | 10070 | 11111 | 11111 |
| **Predicted (LS) mean diff.** | -11503 | 508.7 | -532.6 | 12012 | 10970 | -1041 |
| **SE of diff.** | 2713 | 1982 | 2324 | 2526 | 2802 | 2102 |
| **N1** | 6 | 6 | 6 | 3 | 3 | 10 |
| **N2** | 3 | 10 | 5 | 10 | 5 | 5 |
| **q** | 5.995 | 0.363 | 0.3242 | 6.725 | 5.536 | 0.7006 |
| **DF** | 20 | 20 | 20 | 20 | 20 | 20 |
| **95.00% CI of diff.** | -19098 to -3908 | -5038 to 6055 | -7036 to 5971 | 4941 to 19082 | 3126 to 18814 | -6924 to 4842 |
| **Adjusted P Value** | 0.0021 | 0.9939 | 0.9956 | 0.0006 | 0.0044 | 0.9592 |

$AUC_{inf\_D\_obs}$ in unbound blood in LPS-treated and healthy female (F) and male (M) rats. $AUC_{inf\_D\_obs}$ data are presented in Figure 7 and Table S6.

*S8*

**Table S8. Statistical test details of comparisons of systemic $CL_{\_obs}$ in LPS-treated and healthy female and male rats.**

| Two-way ANOVA and Tukey's multiple comparison test results | | | | | | |
|---|---|---|---|---|---|---|
| **Test details** | **Healthy:F vs. LPS:F** | **Healthy:F vs. Healthy:M** | **Healthy:F vs. LPS:M** | **LPS:F vs. Healthy: M** | **LPS:F vs. LPS:M** | **Healthy: M vs. LPS:M** |
| **Predicted (LS) mean 1** | 95.56 | 95.56 | 95.56 | 49.96 | 49.96 | 104.7 |
| **Predicted (LS) mean 2** | 49.96 | 104.7 | 97.52 | 104.7 | 97.52 | 97.52 |
| **Predicted (LS) mean diff.** | 45.59 | -9.169 | -1.96 | -54.76 | -47.55 | 7.208 |
| **SE of diff.** | 14.72 | 10.75 | 12.6 | 13.7 | 15.2 | 11.4 |
| **N1** | 6 | 6 | 6 | 3 | 3 | 10 |
| **N2** | 3 | 10 | 5 | 10 | 5 | 5 |
| **q** | 4.382 | 1.207 | 0.22 | 5.653 | 4.425 | 0.8944 |
| **DF** | 20 | 20 | 20 | 20 | 20 | 20 |
| **95.00% CI of diff.** | 4.405 to 86.78 | -39.25 to 20.91 | -37.23 to 33.31 | -93.10 to -16.42 | -90.09 to -5.014 | -24.69 to 39.11 |
| **Adjusted P Value** | 0.0267 | 0.8284 | 0.9986 | 0.0036 | 0.025 | 0.9203 |

Clearance ($CL_{\_obs}$) in unbound blood in LPS-treated and healthy female (F) and male (M) rats. $CL_{\_obs}$ data are presented in Figure 7 and Table S6.

*S9*

**Table S9. Statistical test details of comparisons of systemic $t_{1/2}$ in LPS-treated and healthy female and male rats.**

| Two-way ANOVA and Tukey's multiple comparison test results | | | | | | |
|---|---|---|---|---|---|---|
| **Test details** | **Healthy:F vs. LPS:F** | **Healthy:F vs. Healthy:M** | **Healthy:F vs. LPS:M** | **LPS:F vs. Healthy: M** | **LPS:F vs. LPS:M** | **Healthy: M vs. LPS:M** |
| **Predicted (LS) mean 1** | 35.24 | 35.24 | 35.24 | 60.63 | 60.63 | 34.29 |
| **Predicted (LS) mean 2** | 60.63 | 34.29 | 37.26 | 34.29 | 37.26 | 37.26 |
| **Predicted (LS) mean diff.** | -25.39 | 0.9511 | -2.023 | 26.34 | 23.37 | -2.975 |
| **SE of diff.** | 5.046 | 3.536 | 4.249 | 4.907 | 5.444 | 4.083 |
| **N1** | 8 | 8 | 8 | 3 | 3 | 10 |
| **N2** | 3 | 10 | 5 | 10 | 5 | 5 |
| **q** | 7.116 | 0.3804 | 0.6734 | 7.593 | 6.071 | 1.03 |
| **DF** | 22 | 22 | 22 | 22 | 22 | 22 |
| **95.00% CI of diff.** | -39.41 to -11.38 | -8.867 to 10.77 | -13.82 to 9.776 | 12.72 to 39.97 | 8.253 to 38.49 | -14.31 to 8.362 |
| **Adjusted P Value** | 0.0003 | 0.993 | 0.9636 | 0.0001 | 0.0016 | 0.8846 |

Terminal half-life ($t_{1/2}$) in unbound blood in LPS-treated and healthy female (F) and male (M) rats. $t_{1/2}$ data are presented in Figure 7 and Table S6.

## Figures (Supplementary Material)

*Figure S1.*

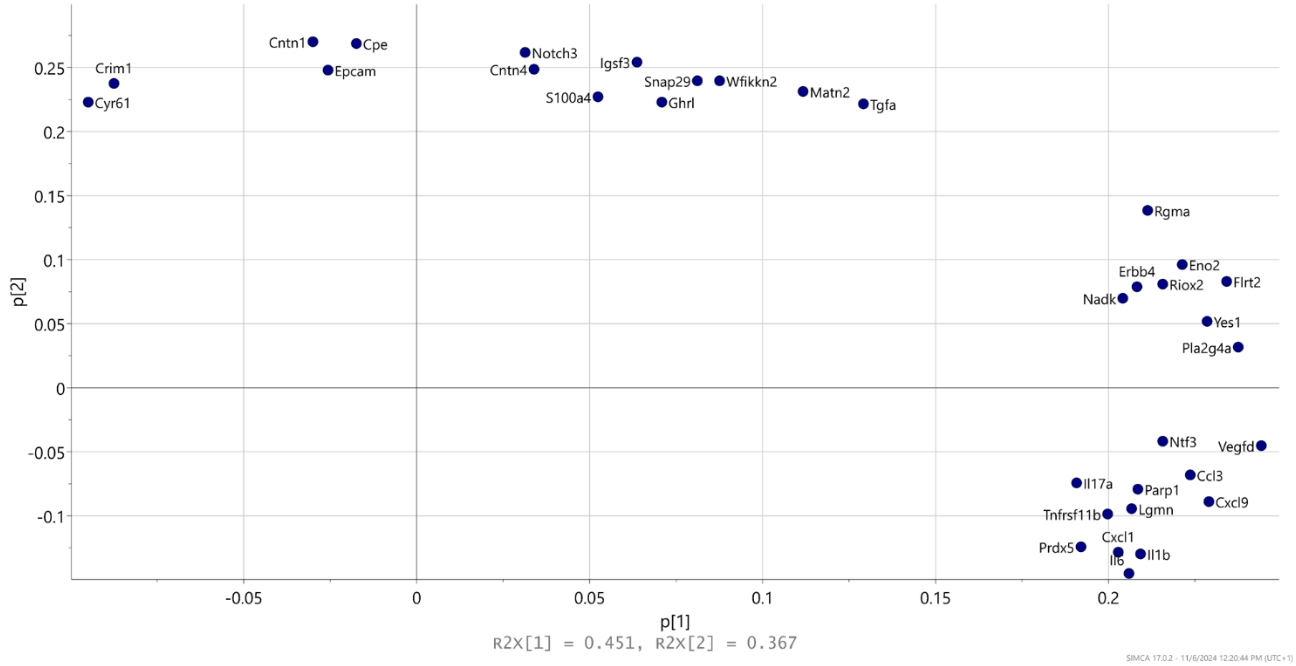


**Figure S1. Loading plot of the first two principal components from the proteomic analysis of plasma samples.** The corresponding PCA score plot is presented in Figure 3 in the main text.

*Figure S2.*

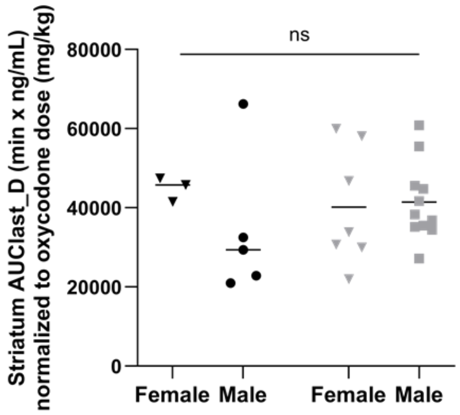


**Figure S2. Similar mean unbound oxycodone exposure in striatum after LPS treatment.** Scatter dot plots comparing unbound oxycodone area under the concentration-time curve (0-240 min) in striatum normalized by oxycodone dose (mg/kg), i.e., $AUC_{last\_D}$ between LPS-treated (black) and healthy (gray) male (dots and squares) and female (triangles) rats. The comparison was performed using an ordinary two-way ANOVA with Tukey's multiple comparison test; ns=not significant.

*Figure S3.*

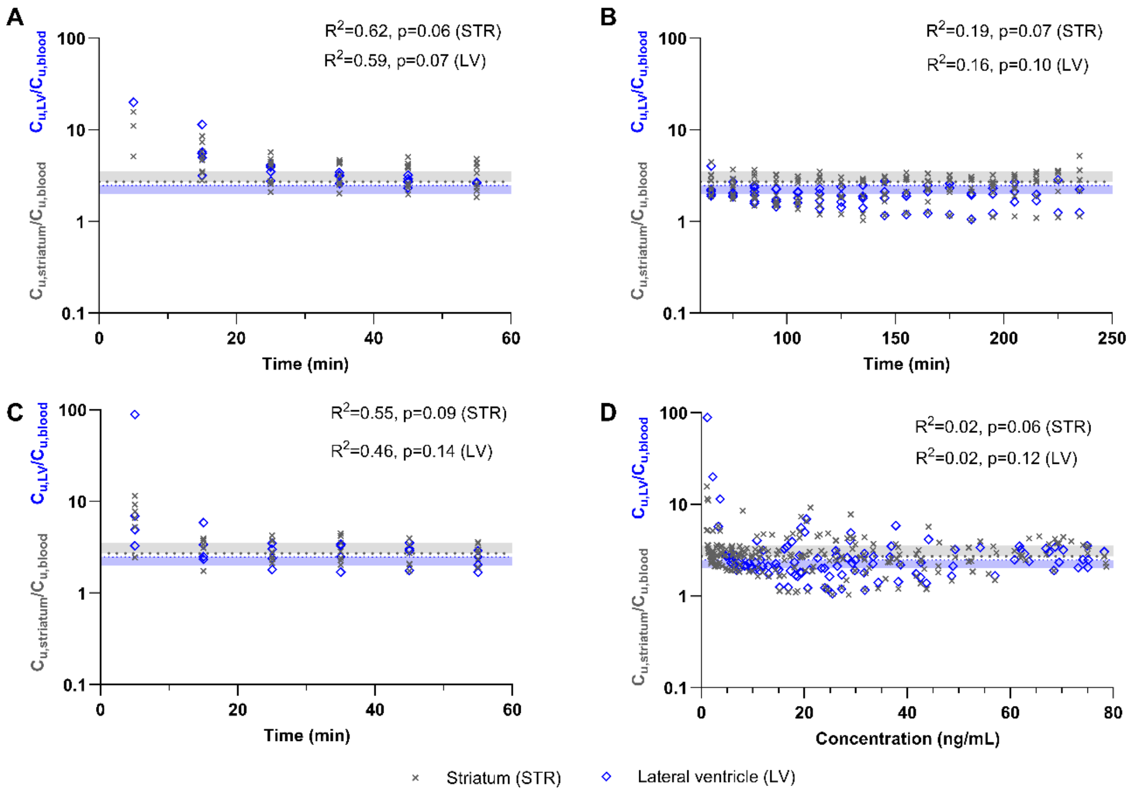


**Figure S3. Unbound concentration ratios in the striatum (STR) and lateral ventricle (LV) versus time and concentration**. Lack of relationships of the single time point ratios of the unbound oxycodone concentration in STR and LV to that in blood ($C_{u,STR}/C_{u,blood}$, gray crosses; $C_{u,LV}/C_{u,blood}$, blue open diamonds), and A) time during infusion (Dosing regimen I, $N_{STR}$=10, $N_{LV}$=6) and B) time after the infusion (Dosing regimen I), C) time during infusion (Dosing regimen II, $N_{STR}$=7, $N_{LV}$=4), and D) unbound blood concentration (Dosing regimen I-II). The horizontal dotted lines represent mean $K_{p,uu}$ in STR (gray) and LV (blue), and the shadowed area represents the respective standard deviation. The goodness of fit is described by the determination coefficients ($R^2$) which were obtained by computing a two-tailed correlation. The goodness of fit, described by the determination coefficient ($R^2$), indicates that neither time nor concentration can explain the variation in the extent of oxycodone CNS disposition described by the unbound concentration ratios.

*Figure S4.*

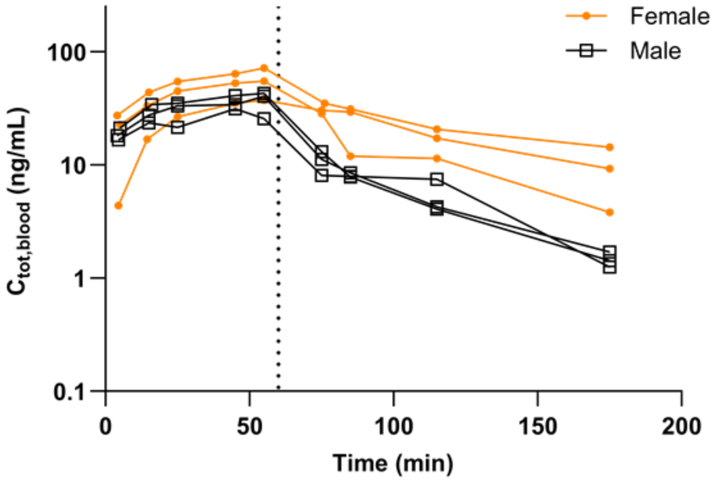


**Figure S4. Total oxycodone concentration-time profiles in blood in LPS-treated female and male rats**. Individual total oxycodone concentration-time profiles in LPS-treated female (orange dots) and male (black squares) rats. The vertical dotted line represents the end of oxycodone infusion at 60 minutes (Dosing regimen I).

## 2. References

1. Krzywinski M, Altman N. Power and sample size. Nature Methods. 2013;10(12):1139-40.DOI: 10.1038/nmeth.2738.
2. Bällgren F, Hammarlund-Udenaes M, Loryan I. Active Uptake of Oxycodone at Both the Blood-Cerebrospinal Fluid Barrier and The Blood-Brain Barrier without Sex Differences: A Rat Microdialysis Study. Pharmaceutical research. 2023;40(11):2715-30.DOI: 10.1007/s11095-023-03583-0.
3. Paxinos G, Watson C. The Rat Brain. 2nd ed. Paxinos G, Watson C, editors: Academic Press; 1986 1986/01/01/. iv p.
4. Jaeger LB, Dohgu S, Sultana R, Lynch JL, Owen JB, Erickson MA, Shah GN, Price TO, Fleegal-Demotta MA, Butterfield DA, Banks WA. Lipopolysaccharide alters the blood-brain barrier transport of amyloid beta protein: a mechanism for inflammation in the progression of Alzheimer's disease. Brain, behavior, and immunity. 2009;23(4):507-17.DOI: 10.1016/j.bbi.2009.01.017.
5. Erickson MA, Hansen K, Banks WA. Inflammation-induced dysfunction of the low-density lipoprotein receptor-related protein-1 at the blood-brain barrier: protection by the antioxidant N-acetylcysteine. Brain, behavior, and immunity. 2012;26(7):1085-94.DOI: 10.1016/j.bbi.2012.07.003.
6. Erickson MA, Hartvigson PE, Morofuji Y, Owen JB, Butterfield DA, Banks WA. Lipopolysaccharide impairs amyloid β efflux from brain: altered vascular sequestration, cerebrospinal fluid reabsorption, peripheral clearance and transporter function at the blood-brain barrier. Journal of neuroinflammation. 2012;9:150.DOI: 10.1186/1742-2094-9-150.
7. Bouw MR, Hammarlund-Udenaes M. Methodological aspects of the use of a calibrator in in vivo microdialysis-further development of the retrodialysis method. Pharmaceutical research. 1998;15(11):1673-9.DOI: 10.1023/a:1011992125204.

8. Boström E, Simonsson US, Hammarlund-Udenaes M. In vivo blood-brain barrier transport of oxycodone in the rat: indications for active influx and implications for pharmacokinetics/pharmacodynamics. Drug metabolism and disposition: the biological fate of chemicals. 2006;34(9):1624-31.DOI: 10.1124/dmd.106.009746.
9. Gustafsson S, Eriksson J, Syvänen S, Eriksson O, Hammarlund-Udenaes M, Antoni G. Combined PET and microdialysis for in vivo estimation of drug blood-brain barrier transport and brain unbound concentrations. NeuroImage. 2017;155:177-86.DOI: 10.1016/j.neuroimage.2017.04.068.
10. Tozer TN. Concepts basic to pharmacokinetics. Pharmacology & therapeutics. 1981;1(12):109-31.DOI: 10.1016/0163-7258(81)90077-2.
11. Gustafsson S, Gustavsson T, Roshanbin S, Hultqvist G, Hammarlund-Udenaes M, Sehlin D, Syvänen S. Blood-brain barrier integrity in a mouse model of Alzheimer's disease with or without acute 3D6 immunotherapy. Neuropharmacology. 2018;143:1-9.DOI: 10.1016/j.neuropharm.2018.09.001.
12. Hu Y, Girdenyté M, Roest L, Liukkonen I, Siskou M, Bällgren F, Hammarlund-Udenaes M, Loryan I. Analysis of the contributing role of drug transport across biological barriers in the development and treatment of chemotherapy-induced peripheral neuropathy. Fluids and barriers of the CNS. 2024;21(1):13.DOI: 10.1186/s12987-024-00519-7.
13. Fridén M, Ljungqvist H, Middleton B, Bredberg U, Hammarlund-Udenaes M. Improved measurement of drug exposure in the brain using drug-specific correction for residual blood. Journal of cerebral blood flow and metabolism : official journal of the International Society of Cerebral Blood Flow and Metabolism. 2010;30(1):150-61.DOI: 10.1038/jcbfm.2009.200.
